# Efficacy and safety of brigatinib in *ALK*-positive non-small cell lung cancer treatment: A systematic review and meta-analysis

**DOI:** 10.3389/fonc.2022.920709

**Published:** 2022-11-03

**Authors:** Puyuan Xing, Xuezhi Hao, Xin Zhang, Junling Li

**Affiliations:** Department of Medical Oncology, National Cancer Center/National Clinical Research Center for Cancer/Cancer Hospital, Chinese Academy of Medical Sciences and Peking Union Medical College, Beijing, China

**Keywords:** non-small cell lung cancer, ALK-positive, brigatinib, efficacy, adverse events

## Abstract

**Background:**

Brigatinib is a central nervous system-active second-generation anaplastic lymphoma kinase (ALK) inhibitor that targets a broad range of *ALK* rearrangements in patients with non-small cell lung cancer (NSCLC). The current study aimed to analyze the pooled effects and adverse events of brigatinib in patients with *ALK*-positive NSCLC.

**Methods:**

The pooled estimates and 95% confidence intervals (CI) were calculated with DerSimonian-Laird method and the random effect model.

**Results:**

The pooled objective response rate (ORR) and disease control rate (DCR) of brigatinib were 64% (95% CI 45%-83%) and 88% (95% CI 80%-96%), respectively. The pooled mPFS was 10.52 months (95% CI 7.66-13.37). In the subgroup analyses by treatment line, the highest mPFS was reached in first-line treatment (24.00 months, 95% CI 18.40-43.20), followed by post-crizotinib second-line treatment (mPFS=16.26 months, 95% CI 12.87-19.65), and second-line with any prior ALK tyrosine kinase inhibitors (mPFS=12.96 months, 95% CI 11.14-14.78). Among patients with any baseline brain metastases, the pooled intracranial ORR (iORR) was estimated as 54% (95% CI 35%-73%) for any treatment line, and 60% (95% CI 39%-81%) for first-line treatment. Intracranial PFS (iPFS) reached 19.26 months (95% CI 14.82-23.70) in patients with any baseline brain metastases. Creatine phosphokinase (CPK) increased (44%, 95% CI 26%-63%), diarrhea (37%, 95% CI 27%-48%), and nausea (28%, 95% CI 17%-39%) of any grade were the most common adverse events.

**Conclusion:**

Brigatinib is effective in the treatment of patients with *ALK*-positive NSCLC, particularly showing robust intracranial PFS. Brigatinib used as first-line treatment yielded superior PFS compared with brigatinib used as other treatment lines. These results suggested a benefit of using brigatinib earlier in the patient’s management. All adverse events are manageable, with CPK increased and gastrointestinal reactions found to be the most common types.

**Systematic Review Registration:**

https://inplasy.com/inplasy-2022-3-0142/, identifier (INPLASY202230141).

## 1 Introduction

Non-small cell lung cancer (NSCLC) accounts for approximately 80-85% of lung cancer cases, which are the most common fatal malignancy and leading cause of cancer mortality worldwide (1). Unfortunately, the prognosis of NSCLC remains poor, with estimated 5-year survival rate of 16%, and more than 50% of patients have advanced disease at diagnosis. For patients with advanced NSCLC, platinum-based chemotherapy is the standard treatment. For these patients, objective response rate (ORR) was approximately 30%; however, the therapeutic effect generally lasts only 4-5 months (2–4). Fortunately, with the increasing understanding of the pathogenesis of NSCLC in the past decades, the prognosis of patients has been improved substantially by using newly developed targeted drugs (5, 6). Anaplastic lymphoma kinase (ALK) gene rearrangement accounts for approximately 3-5% of advanced NSCLC (7). Advanced NSCLC harboring an *ALK* rearrangement (*ALK*-positive NSCLC) can be effectively treated with small-molecule tyrosine kinase inhibitors (TKIs) that target ALK, which have shown stunning efficacy and favorable safety profile in this subgroup of patients (8).

Crizotinib was the first ALK-TKI approved for *ALK*-positive NSCLC by the U.S. Food and Drug Administration (FDA). In first-line treatment, crizotinib achieved ORR from 61 to 74% with a median progression-free survival (PFS) of 8–11 months (9–11). However, almost all patients with *ALK*-positive NSCLC treated with crizotinib eventually develop resistance, leading to disease progression, including the development of central nervous system (CNS) metastases (12–14). Several next-generation ALK-TKIs have been developed including second-generation TKIs such as ceritinib, alectinib, and brigatinib (15–17). These next-generation ALK-TKIs have been proved to be more potent and CNS–penetrant compared to crizotinib and can retain variable activity against different crizotinib-resistant *ALK* mutations (18, 19).

Brigatinib is a new second-generation ALK inhibitor that was developed to overcome resistance to crizotinib. In a multi-center phase II study, brigatinib showed strong effectiveness among patients with crizotinib-refractory *ALK*-positive NSCLC. Among 222 patients receiving one of two dosing regimens of brigatinib (90 mg once daily versus 180 mg once daily with a 7-day lead-in at 90 mg), the confirmed ORRs were reported to be 45% and 54%, with a median PFS of 9.2 months and 16.7 months, respectively (20). Based on findings reported in this phase II study, the U.S. FDA granted accelerated approval to brigatinib in patients with locally advanced or metastatic *ALK*-positive NSCLC who have progressed on or are intolerant to crizotinib in April 2017. Further in May 2020, U.S. FDA also issued full approval for brigatinib for front line treatment. Since first approval of brigatinib, studies have been conducted in clinical and real-world settings that evaluated efficacy and safety of brigatinib in different countries. However, substantial differences have been observed in regard to clinical outcomes, which might be partly attributed to small sample size, variances in patient characteristics and study settings. For example, the ORRs ranged from 0.40 to 0.97 in two recent clinical studies (21, 22). Hence, it is of utmost importance to calculate the pooled effect of brigatinib in order to clarify its efficacy.

In the current study, we conducted a systematic review and meta-analysis to investigate the efficacy and adverse events of brigatinib among patients with *ALK*-positive NSCLC in both clinical and real-world settings. The findings of this study shall enlighten further scientific research and clinical applications.

## 2 Methods

### 2.1 Search strategy

We identified eligible studies through a comprehensive search of PubMed (Medline), EMBASE (Excerpta Medica Database), Cochrane Library and Web of Science up to August 2021. Keyword search terms were (‘brigatinib’) and (‘non-small cell lung cancer’ or ‘NSCLC’). We have also inspected the reference list of the retrieved studies in case we would miss relevant studies which met our inclusion criteria. Additionally, in order to obtain the latest information, conference abstracts that were presented in the 57th Annual Meeting (Virtual) of the American Society of Clinical Oncology (ASCO) June 4–8, 2021, and European Society for Medical Oncology (ESMO) Congress September 16-21, 2021 were also screened.

### 2.2 Selection criteria

**Figure 1:**
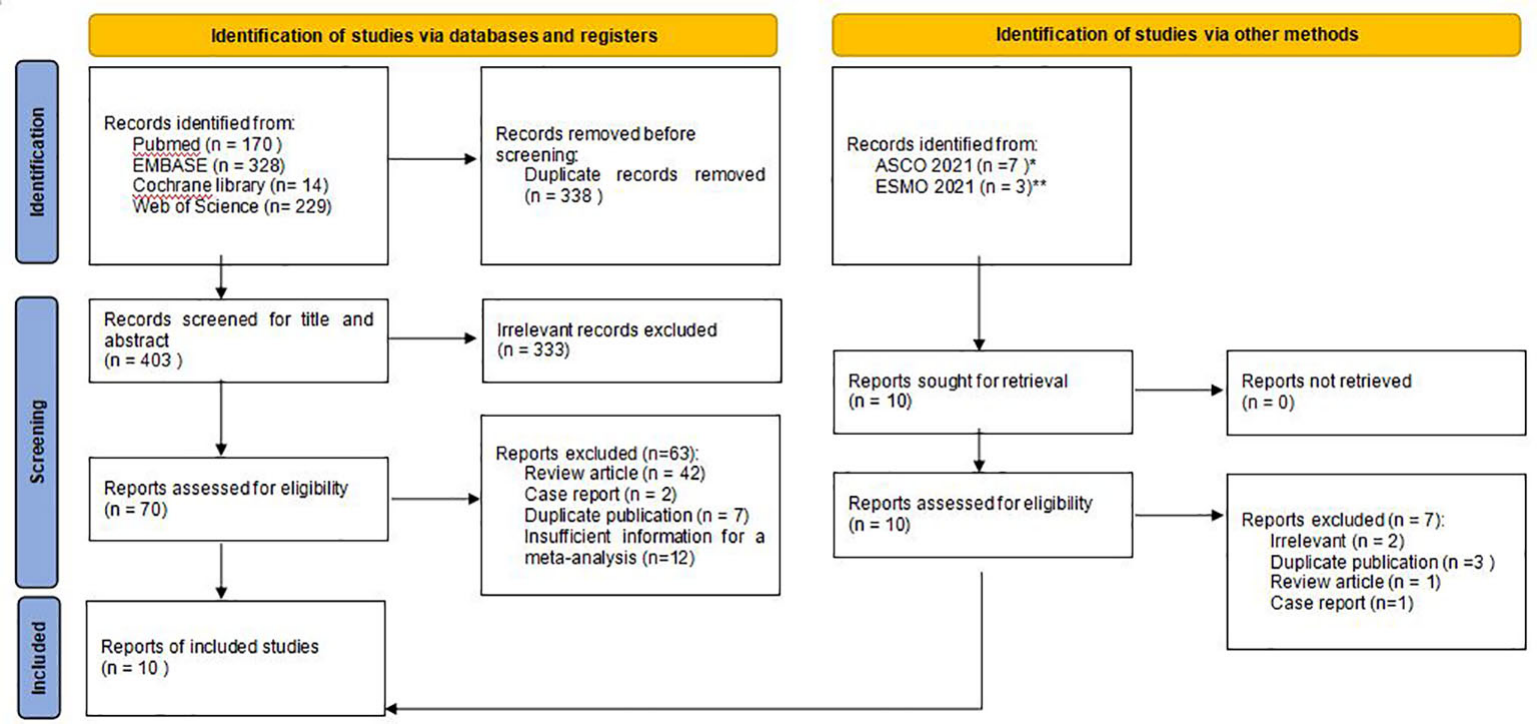
Flow chart of study selection. ASCO*: American Society of Clinical Oncology; ESMO**: European Society for Medical Oncology.

Eligible studies were selected based on prespecified PICOS criteria. P (participants): *ALK*-positive NSCLC; I (intervention): oral brigatinib therapy; C (control): none; O (outcomes): ORR, disease control rate (DCR), PFS, intracranial ORR (iORR), intracranial PFS (iPFS), or adverse events (AEs); S (study designs): phase I, II or III clinical study, prospective cohort study, retrospective cohort study, or real-world evidence study. Articles dealing with mechanism research, pharmacology research, other non-efficacy research, or those not in English were excluded. We did not exclude studies involving patients pretreated with prior ALK inhibitors, nor did we exclude studies involving patients receiving chemotherapy. Where there were duplicate studies, articles published earlier or those that provided more detailed information or with longer follow-up time were selected (**Figure 1**). Two independent reviewers screened the articles according to the criteria to determine eligibility, and a third researcher resolved the differences if any.

### 2.3 Data extraction and analysis

This study conducted data analysis according to the PRISMA Statement (23). The following information was extracted in a predesigned form: first author, publication year, study design, population, age, male percentage, sample size, country, follow-up time, brigatinib medication, brain metastases at diagnosis, previous use of ALK-TKI prior to brigatinib, ORR, DCR, PFS, iORR, iPFS, and AEs. One researcher was responsible to extract the data independently, whereas another reviewed the data to ensure accuracy.

### 2.4 Statistical methods

To evaluate the therapeutic effect of brigatinib in patients with *ALK*-positive NSCLC, we analyzed the best responses. The estimated odds ratio/percentage/months and 95% confidence interval (95% CI) of the ORR, DCR, PFS, iORR, and iPFS were extracted. In cases when multiple sets of data were provided in a study, we extracted only the best response data using standard dosage treatment (180 mg qd with 7-day 90 mg lead-in).

The toxicities and AEs reported in each study were classified and merged. Only the incidence of 10 common AEs was analyzed and reported. Stata 14 was used for data merger analysis and heterogeneity tests. Heterogeneity among the studies was assessed by the Cochran Q test and the I^2^ statistics. For the Q statistic, *P* < 0.10 was considered statistically significant for heterogeneity. For the I^2^ statistic, which indicates the percentage of the observed between-study variability due to heterogeneity rather than chance, the following ranges were used: no heterogeneity (I^2^ = 0%–25%), low heterogeneity (I^2^ = 25%–50%), moderate heterogeneity (I^2^ = 50%–75%), and high heterogeneity (I^2^ = 75%–100%). DerSimonian-Laird method and the random effect model were used to calculate pooled effect size and draw forest plots. For studies with moderate or higher heterogeneity (I^2^ ≥50%, P<0.10), we also conducted meta-regression to analyze the sources of heterogeneity in the studies. Finally, sensitivity analysis was also conducted in order to explore the impact of excluding an individual study on the pooled results. Two-tailed *P* value < 0.05 was defined as with statistical significance for all tests, except for heterogeneity test between studies.

The protocol of this study has been registered in INPLASY (ID: INPLASY202230142).

### 2.5 Quality assessment

As only single-arm cohort studies were included in the final analysis, CASP-Cohort-Study-Checklist was used for quality assessment (24). The CASP-Cohort List, a quality assessment tool, was proposed by the Oxford Evidence-based Medical Center in 2004 for cohort studies. The tool consists of 12 questions and 3 sections which were used to evaluate each study.

### 2.6 Assessment of publication bias

Stata 14 with meta-regression was used to analyze the sources of heterogeneity. Publication bias was inspected by a Deeks funnel plot. In addition, Begg’s and Egger’s test was also conducted to testify the funnel plot asymmetry.

## 3 Results

### 3.1 Eligible studies

We retrieved 741 articles from 4 databases in the initial search. After reading the title and abstract, excluding duplicate and irrelevant articles, we selected 70 articles for further review. After manual reading of the full text, 62 papers were excluded due to the following reasons: review article (n=42), case report (n=2), duplicate publication (n=6), or insufficient information for a meta-analysis (n=12). In addition, 3 conference abstracts with most updated results (ALTA, BrigALK2, J-ALTA) were also included after searching and reading from abstracts presented in ASCO 2021 (n=2), and ESMO Congress 2021 (n=1) (22, 25, 26). Finally, 10 articles with 942 patients were included in this meta-analysis (**Figure 1**) (21, 22, 25–33).

### 3.2 Study characteristics and quality evaluation

Baseline features of each included study are shown in **Table 1**. The final analysis included 10 studies that consisted of a total sample size of 942, including six randomized clinical trial studies and four retrospective real-world evidence studies. The 10 studies were first published in 2018 and most recently in 2021. The sample size ranged from 20 to 301, covering Asia, Europe, the Americas, and other regions. The median age ranged between 43-61 years. Male patients accounted for 41% to 60%, and 17% to 82% of the included subjects had brain metastases at baseline. Only two studies used brigatinib as first-line treatment. The details about treatment lines and number of participants for each study are also presented in **Supplemental Table 1**.

**Table 1:** Characteristics of the 10 included studies.

Study	Study design	Population	Age (years)	Male%	Sample size	Country	Follow-up(months)	Brigatinib dose	Brain metastases at diagnosis	ALK-TKI before brigatinib
Camidge 2018 (32)	Single-arm, open-label, multicenter study; phase II, open-label, multicenter study	PhI/II (Phase 1/2 trial (NCT01449461). Age ≥ 18 years, adequate organ and hematologic function, and one or more measurable lesions.	53 (30-73)	52%	50	USA and Spain	24.9 (0.2-47.6)	90-240 mg/day	100%	ALK-TKI naive or pretreated
Lin 2018 (28)	Multicenter retrospective study	Patients were identified at three participating institutions. All patients had advanced NSCLC with an *ALK* rearrangement. Patients had to have received alectinib with progression of disease before receiving brigatinib.	55 (22-76)	41%	22	USA	–	NA	18 (82%)	ALK-TKI pretreated: 1: 5 (23%); 2: 15 (59%); 3: 4 (18%);
Heredia 2020 (31)	Retrospective observational study	Patients ≥18 years of age with a pathologically confirmed diagnosis of locally advanced or metastatic disease (stage IIIB–IV) NSCLC, *ALK* positive and progression after at least one prior ALK-TKI therapy or treatment discontinuation due to intolerable toxicity.	53.43 (27–73)	56.5%	46	America	9.3 (0.26–28.39)	180 mg qd with 7-day 90 mg lead-in	25 (54.3%)	ALK-TKI pretreated
Descourt 2021 (26)	Retrospective multicentric study (BrigALK2)	Inclusion criteria were: at least 18 years old; advanced NSCLC; *ALK* positive NSCLC; previous treatment with at least one ALK inhibitor including crizotinib.	60 ± 12.7	40.4%	183	France	40.5 (38.4-42.4)	180 mg qd with 7-day 90 mg lead-in	131 (71.1%)	ALK-TKI pretreated
Camidge 2021 (27)	Phase III, open-label, randomized study (ALTA-1L)	Adults with locally advanced/metastatic NSCLC and ≥ 1 measurable lesion who had not received prior *ALK*-targeted therapy. Asymptomatic or stable CNS metastases were permitted.	58 (27-86)	50%	137	20 countries	40.4 (0-52.4)	180 mg qd with 7-day 90 mg lead-in	47 (34.1%)	ALK-TKI naive
Nishio 2021 (29)	Single-arm, multicenter, open-label study (J-ALTA)	Eligible patients (≥20 years of age) confirmed stage IIIB, stage IIIC, or stage IV NSCLC with documented *ALK* rearrangement.	53 (23–82)	47%	47	Japan	12.4	180 mg qd with 7-day 90 mg lead-in	8 (17.0%)	ALK-TKI pretreated
Stinchcombe 2021 (21)	Single arm phase 2 trial (NCT02706626)	Patients were required to have advanced *ALK* + NSCLC, progression on a next generation ALK TKI, ECOG performance status of 0-2, adequate organ function, and measurable disease. There was no restriction on the number of prior therapies.	55 (32-71)	60%	20	USA	22 (0.89-30.5)	180 mg qd with a 7-day lead-in at 90 mg	11 (55%)	ALK-TKI pretreated
Popat 2021 (30)	Retrospective chart review (UVEA-Brig)	Adults with *ALK*-positive mNSCLC, including those with brain lesions, resistant to or intolerant of ≥1 prior ALK inhibitor and ECOG performance status ≤3 were eligible.	53 (29–80)	43%	104	Austria, France, Germany, Ireland, Italy, Spain, Norway, Switzerland, UK	16.5	89.4% received standard dose	66 (63%)	ALK-TKI pretreated
Gettinger 2021 (25)	Single -arm, open-label, multicenter study (Phase I/II); phase II, open-label, multicenter study (ALTA)	Phase I/II (NCT01449461) was a single arm trial with nine sites in the United States and Spain, and ALTA (NCT02094573) was a randomized phase II trial with 71 sites in 18 countries. In both trials, eligibility stipulated age ≥ 18 years, adequate organ and hematologic function, and one or more measurable lesions.	Phase I/II 54 (29,83) Arm A 51 (18,82) Arm B 57 (20,81)	Phase I/II: 51%; ALTA: Arm A 45%; Arm B 42	Phase I/II: 79; ALTA: Arm A: 112; Arm B: 110	20 countries	Phase I/II 27.7 (0.2,88.3); ALTA: Arm A 19.6 (0.1,62.8); Arm B 28.3 (0.1, 66.8)	180 mg qd with a 7-day lead-in at 90 mg	Phase I/II study: 63%; ALTA: 67% (arm B)	ALK-TKI naive or pretreated
Kondo 2021 (22)	Phase 2, single-arm, open-label, multicenter study (J-ALTA)	Adults (aged ≥20 y), stage IIIB/IIIC/IV *ALK*+ NSCLC. TKI-naive.	61 (29,82)	47%	32	Japan	14.2 (3,19)	NA	7 (22%)	ALK-TKI naive

NA, Not Available.

The result of literature quality assessment is shown in Appendix 1. Detection bias was moderate as only 5 studies (50%) used an independent review committee (IRC) to assess disease progression or treatment response.

### 3.3 Meta-synthesis of results

**Figure 2:**
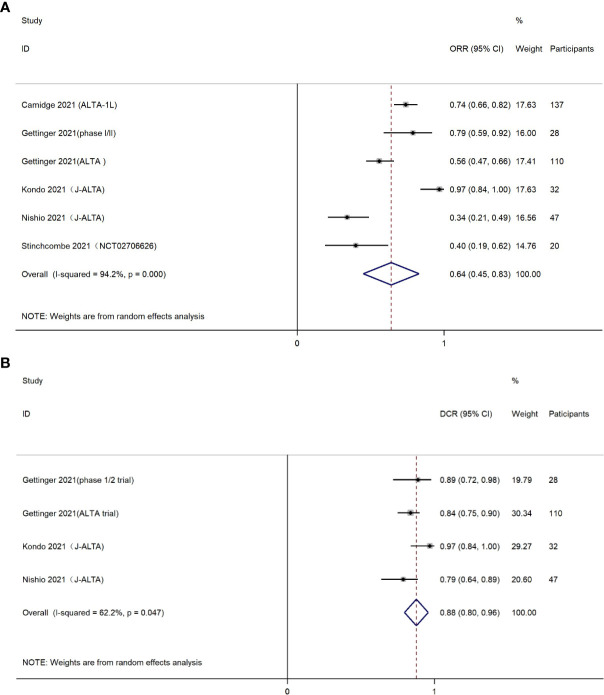
**(A)** Forest plot of objective response rate (ORR); **(B)** Forest plot of disease control rate (DCR).

Six studies reported results of ORR. The ORR in the combination group was 64% (95% CI, 45%-83%), but large heterogeneity of the overall ORR was observed, which was statistically significant (I^2^ = 94.2%, P<0.001) (**Figure 2A**). It is worth mentioning that brigatinib was used as first-line treatment in two studies with a total of 169 patients (22, 27). The subgroup analysis indicated a higher ORR of 86% (95% CI, 63%-108%) among patients who received brigatinib as first-line treatment (**Supplemental Figure 1**).

The DCR was presented in four eligible studies, containing 35 patients treated with brigatinib as first-line drug, and 182 patients treated with brigatinib as second-line or higher line drug. The pooled DCR was estimated as 0.88 (95% CI, 0.80-0.96) (**Figure 2B**). Chi-square test and I^2^ statistic demonstrated the statistical heterogeneity (I^2^ = 62.2%, *P*=0.047), indicating moderate heterogeneity in the overall DCR.

**Figure 3:**
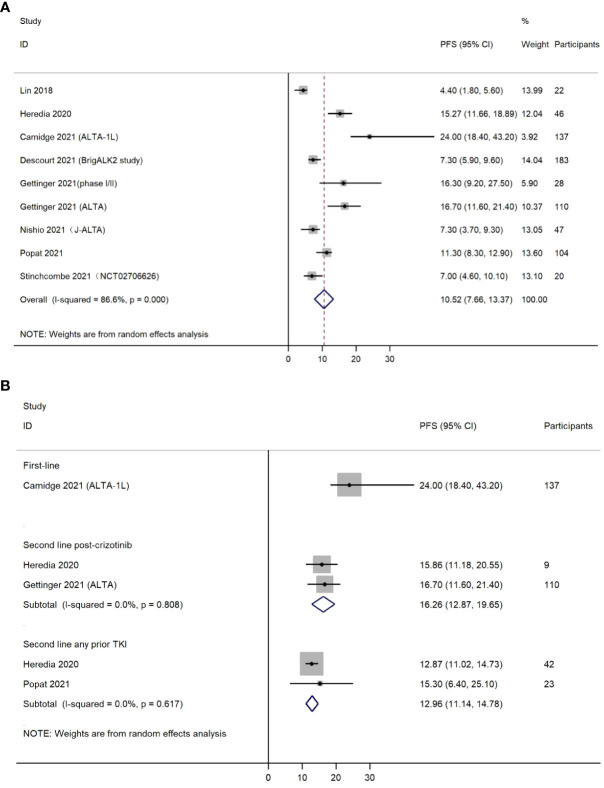
**(A)** Forest plot of overall progression free survival (PFS); **(B)** Forest plot of progression free survival (PFS) by treatment lines.

Nine included studies reported PFS. It should be mentioned that these nine studies did not completely overlap with the six studies included in the analysis for ORR. The reason was that some of the six studies provided both ORR and PFS, whereas others only provided ORR or PFS. The pooled PFS was 10.52 months (95% CI, 7.66-13.37) (**Figure 3A**). Cochran’s Q and I^2^ statistics showed moderate level of heterogeneity with statistical significance (I^2^ = 86.6%, *P*<0.001). Subgroup analyses based on different treatment lines were also performed for PFS (**Figure 3B**). Only one study (n=137) used brigatinib as first-line treatment, providing a median PFS of 24.00 months (95% CI, 18.40-43.20). Two studies (n=119) investigated efficacy for brigatinib as second-line medication post crizotinib (PFS=16.26 months, 95% CI, 12.87-19.65). Two other studies (n=65) were conducted among NSCLC patients using brigatinib as second-line treatment after use of any prior TKI (PFS=12.96 months, 95% CI 11.14-14.78).

**Figure 4:**
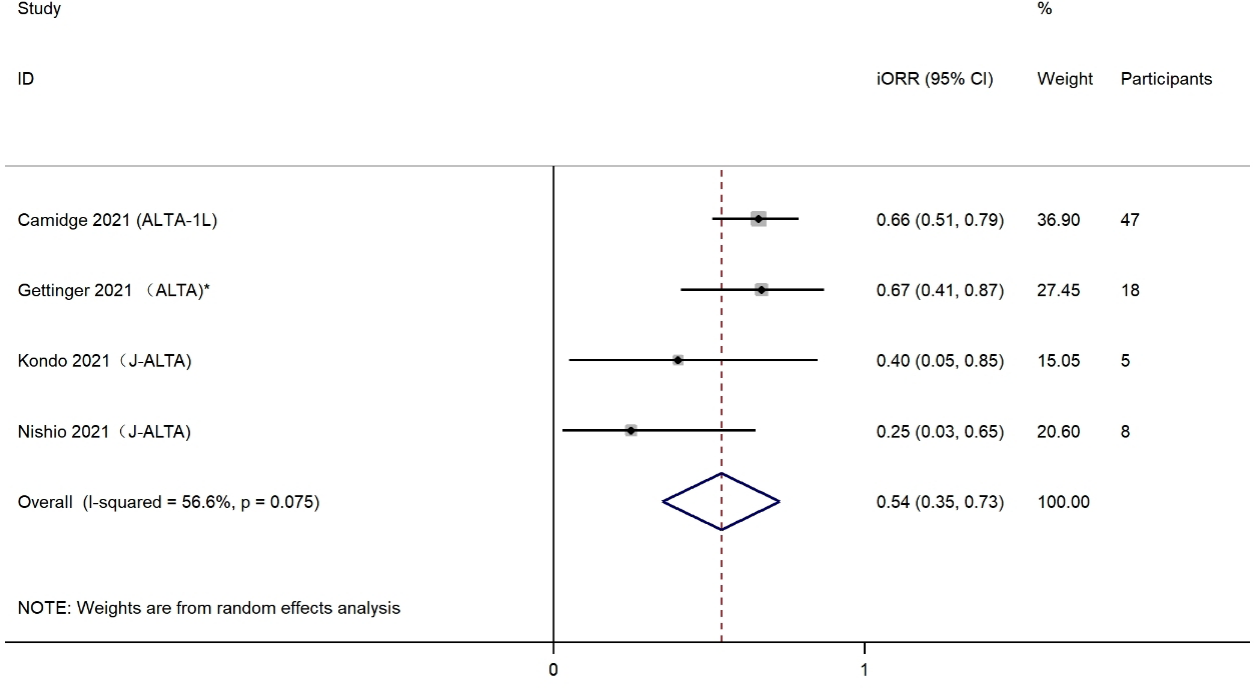
Forest plot of intracranial objective response rate (iORR).

The iORR was presented in four eligible studies, including 78 patients with any baseline brain metastases or measurable CNS metastases. The effects of brigatinib treatment on iORR are shown in **Figure 4**. Estimations of individual iORR ranged from 25% to 66%, which resulted in a summary iORR of 54% (95% CI: 35%-73%). Moderate heterogeneity was detected (I^2^ = 56.6%, *P*=0.075) and a random effect model was selected to summarize effect size. Subgroup analysis was also conducted to evaluate the iORR efficacy of brigatinib when used as first-line treatment. Analysis of two studies with a total of 52 patients indicated a higher iORR of 60% (95% CI, 39%-81%) among patients who received brigatinib as first-line treatment (**Supplemental Figure 2**).

**Figure 5:**
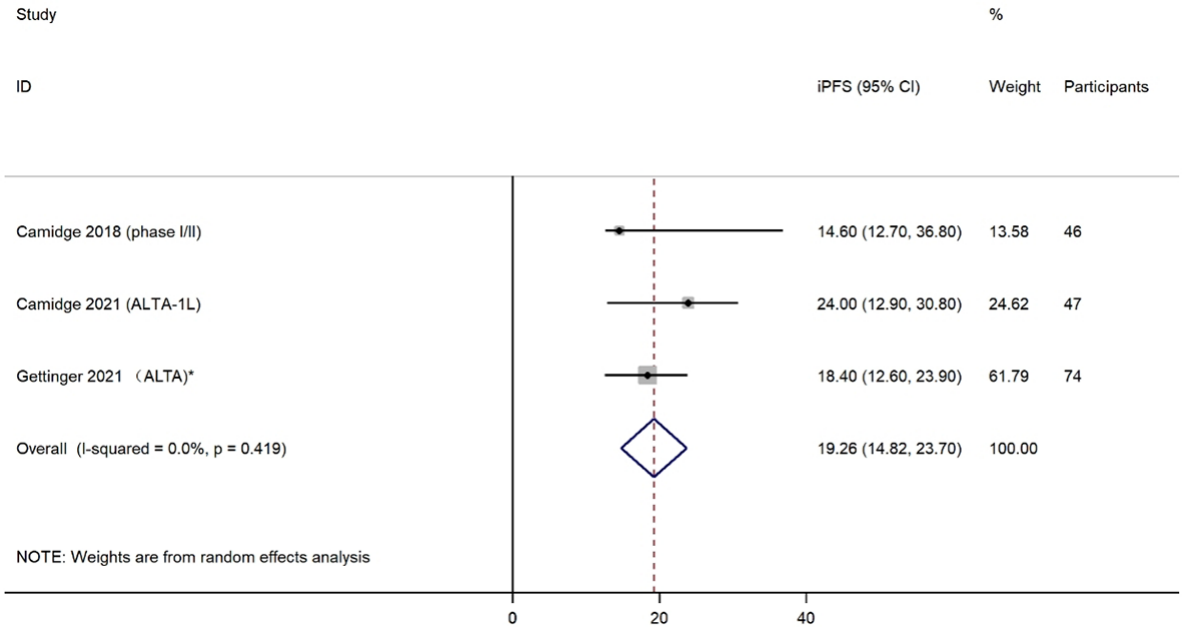
Forest plot of intracranial progression free survival (iPFS).

Three studies reported iPFS among a total of 167 patients with any baseline brain metastases. The effects of brigatinib treatment on iPFS are shown in **Figure 5**. Median iPFS ranged from 14.60 months to 24.00 months. The pooled iPFS was 19.26 months (95% CI: 14.82-23.70). No heterogeneity was detected based on testing for included studies (I^2^ = 0.0%, *P*=0.419).

### 3.3.1 Sensitivity analysis

Sensitivity analysis was conducted for studies that showed moderate or high heterogeneity. Results are shown in **Supplemental Figure 3**. Results showed that the study by Kondo et al. has the greatest impact on summary results of ORR (**Supplemental Figure 3A**). After excluding the study by Kondo et al., the summary ORR was 0.57 (95% CI 0.41-0.73) (data not shown). All 95% CI of ORR in the sensitivity analysis ranged between 0.36-0.88.

For sensitivity analysis of DCR, the study by Kondo et al. also showed the greatest impact on pooled effect size (**Supplemental Figure 3B**). After excluding the study by Kondo et al., the summary DCR was 0.83 (95% CI 0.78-0.90) (data not shown). The range of 95% CI in sensitivity analysis was 0.77-1.00.

### 3.3.2 Source of heterogeneity

First, we performed a meta-regression analysis of the ORR. Sample size was used as a covariate to perform single factor meta-regression analysis (*P*=0.925) (**Supplemental Figure 4A**). Sample size did not contribute to heterogeneity. Subsequently, we also used brain metastases at baseline as a single covariate to conduct univariate meta-regression analysis. Baseline brain metastases had no significant effect on heterogeneity either (*P*=0.890) (**Supplemental Figure 4B**).

We also performed meta-regression analysis for both DCR and PFS. However, both sample size and baseline brain metastases were not contributors to heterogeneity for these two outcome effects (**Supplemental Figures 5**, **6**). Because of incomplete data collection of study factors, it is difficult to identify the sources of heterogeneity.

### 3.4 Assessment of AEs

**Figure 6:**
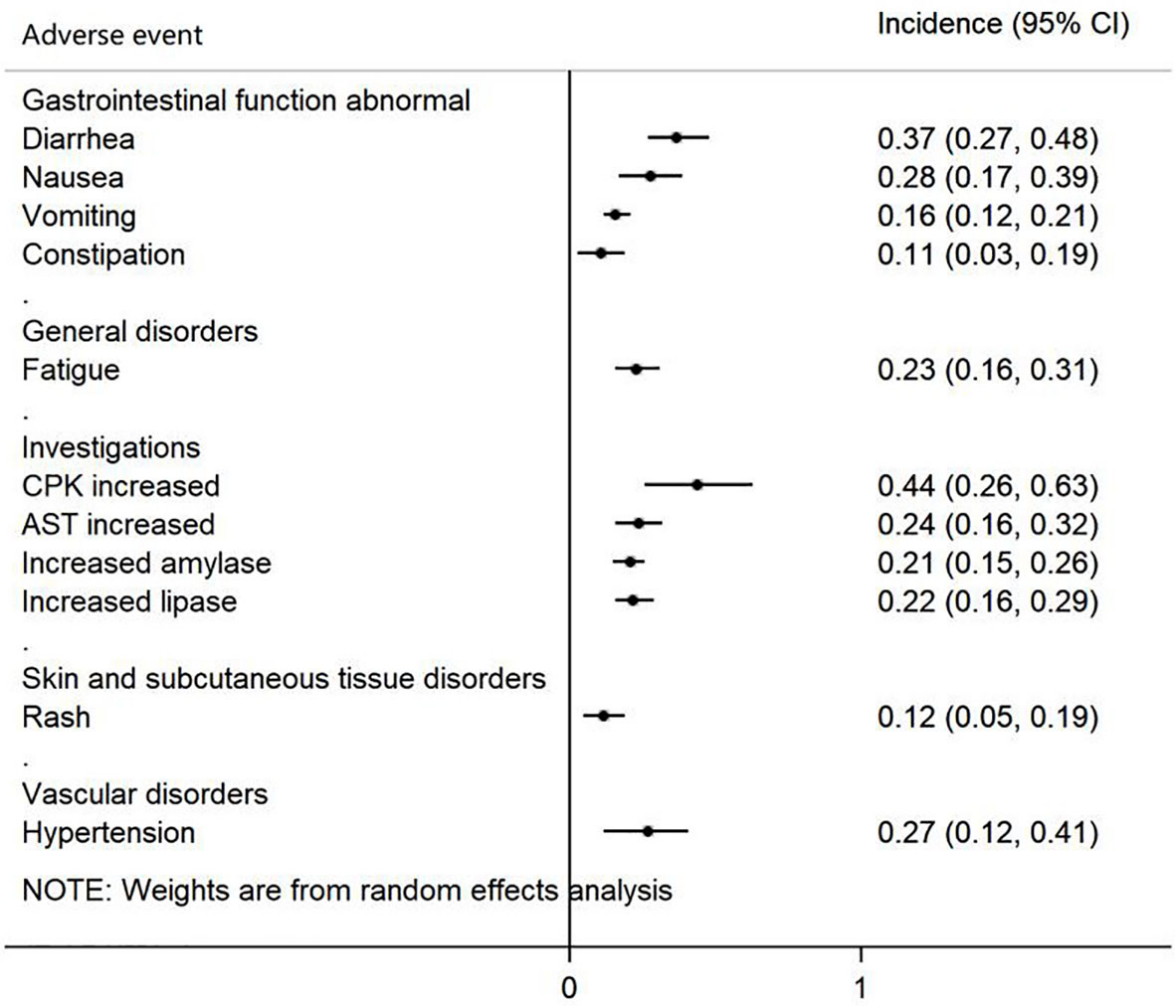
Forest plot of adverse events (AEs).

A total of 7 studies provided data on AEs, which reported 54 AEs. Because different studies may have different descriptions of the same AEs and the classification of AEs is different, we reclassified 11 AEs, which were mentioned in at least five studies (**Table 2**). The Forest plot is shown in **Figure 6**, in which AEs were shown based on the following five groups: 1) gastrointestinal function abnormal; 2) general disorders; 3) investigation; 4) skin and subcutaneous tissue disorder; and 5) vascular disorders. Creatine phosphokinase (CPK) increased, diarrhea, and nausea were the three most common AEs and occurred in 44% (95% CI 26-63%), 37% (95% CI 27-48%) and 28% (95% CI 17-39%) of patients, respectively.

**Table 2:** Summary of toxicity.

Toxicity	Classification	Incidence	95% CI	Studies included
Diarrhea	Gastrointestinal function abnormal	0.37	0.27-0.48	7
Nausea	Gastrointestinal function abnormal	0.28	0.17-0.39	7
Vomiting	Gastrointestinal function abnormal	0.16	0.12-0.21	6
Constipation	Gastrointestinal function abnormal	0.11	0.03-0.19	5
Fatigue	General disorders	0.23	0.16-0.31	5
CPK increased	Investigations	0.44	0.26-0.63	7
AST increased	Investigations	0.24	0.6-0.32	5
Increased amylase	Investigations	0.21	0.15-0.26	7
Increased lipase	Investigations	0.22	0.16-0.29	7
Rash	Skin and subcutaneous tissue disorders	0.12	0.05-0.19	5
Hypertension	Vascular disorders	0.27	0.12-0.41	6

### 3.5 Assessment of publication bias

Funnel plot of DCR and PFS showed asymmetry, while ORR, iORR, iPFS did not show asymmetry (**Supplemental Figure 7**). Statistical tests for funnel plot asymmetry, both Begg’s test and Egger’s test, did not detect statistically significant asymmetry for all effect size evaluated, except iPFS (*P*=0.022) (**Supplemental Table 2**).

## 4 Discussion

### 4.1 Summary of the findings

The current meta-analysis included 10 articles consisting of 6 clinical trials and 4 real-world evidence studies. Data from 942 patients were analyzed. The ORR and DCR of patients with *ALK*-positive NSCLC were 0.64 (95% CI 0.45-0.83) and 0.88 (95% CI 0.80-0.96), respectively, and the PFS was 10.52 months (95% CI 7.66-13.37). In subgroup analyses by treatment line, brigatinib used as first-line treatment showed the longest median PFS (24.00 months, 95% CI 18.40-43.20). For intracranial efficacy, the pooled iORR was 0.54 (95% CI 0.35-0.73), while iPFS reached 19.26 months (95% CI 14.82-23.70). CPK increased, diarrhea, and nausea were the most common AEs of any grade. These results indicate that brigatinib is effective in the treatment of patients with *ALK*-positive NSCLC, particularly showing robust intracranial PFS. Brigatinib used as first-line treatment yielded superior PFS compared with brigatinib used as other treatment lines. All adverse events are manageable, with gastrointestinal reactions and CPK increased found to be the most common types.

### 4.2 Comparisons with other ALK inhibitors

In the last decade, the treatment of advanced NSCLC has shifted into determining molecular subtypes of the disease based on oncogenic drivers, which has led to the introduction of several newly approved biological agents (33). Numerous systematic review and meta-analysis studies have estimated the efficacy of other second generation ALK inhibitors such as alectinib and ceritinib. However, studies on brigatinib are scarce. A network meta-analysis study presented in the 2020 World Conference on Lung Cancer (WCLC 2020, Singapore) compared the efficacy of brigatinib with other approved ALK inhibitors or chemotherapy in patients with locally advanced or metastatic ALK inhibitor-naïve *ALK*-positive NSCLC (34). Five global RCTs (ALEX, ALTA-1L, ASCEND-4, PROFILE 1007, PROFILE 1014) evaluating 4 ALK inhibitors (alectinib, brigatinib, ceritinib, crizotinib) as first-line treatment in *ALK*+ NSCLC were included in the final analysis (34). This study found that 1L brigatinib had superior effects on IRC-assessed PFS compared to crizotinib (HR=0.49, 95% CI 0.35-0.68), and ceritinib (HR=0.42, 95% CI 0.26-0.67), while no significant differences were observed between brigatinib and alectinib (34). These results were in line with a more recent network meta-analysis study by Chuang et al., who updated the efficacy comparisons based on the most recent results of phase II-III clinical trials (CROWN, ALTA-1L, ALEX, J-ALEX, ALESIA, eXalt3). In this study, Chuang and his colleagues confirmed the superiority of brigatinib over crizotinib in terms of PFS (HR=0.49, 95% CI 0.35-0.69). Specifically, brigatinib showed stronger efficacy in patients with baseline brain metastasis (HR=0.25, 95% CI 0.14-0.44) compared to crizotinib, and the KM-estimated 4-year OS rate was 71% (53%-83%) with brigatinib (35). It is worth mentioning that the ALTA-1L study also confirmed that brigatinib could exhibit superior efficacy compared with crizotinib regardless of *EML4-ALK* variant and *TP53* mutation (27). Although low- (300 mg twice daily) and high-dose (600 mg twice daily) of alectinib showed lower HR than brigatinib in pairwise comparisons for PFS, no significant differences were observed (35). Moreover, Chuang et al. found that lorlatinib had a noticeable benefit over brigatinib in both overall PFS (HR=0.57, 95% CI 0.34-0.95) and non-brain metastases-PFS (HR=0.49, 95% CI 0.27-0.91) (35). However, ORR did not differ between brigatinib and lorlatinib. A recent French cohort study, LORLATU, investigated the efficacy and safety of lorlatinib after the failure of at least one ALK-TKI in *ALK*-positive NSCLC (36). The use of lorlatinib in this setting yielded an ORR of 49% and a median PFS of 9.9 months. Findings from this study confirm the position of lorlatinib as an effective rescue treatment after resistance to first- and second-generation ALK-TKIs, and the optimal sequencing of ALK-TKIs still remains to be further analyzed.

Of note, data on median OS are often unavailable in current studies. Our current study included the final results of the ALTA-1L trial, with approximately 15 months of additional follow-up since the second interim analysis (median follow-up=40 months for brigatinib) (27). However, OS was still maturing at final analysis (30% event rate) and indicated similar OS in the brigatinib and crizotinib arms (HR=0.81, 95% CI, 0.53-1.22). It is worth mentioning that, in this largest RCT comparing the efficacy of brigatinib and crizotinib, a cross-over design has been assigned. A total of 65 patients in the crizotinib arm crossed over to brigatinib after BIRC-assessed progression (after 10-day washout from crizotinib). The 3-year OS was 71% (95% CI, 62%-78%) in the brigatinib arm, and 68% (95% CI, 59%-75%) in the crizotinib arm without adjustment for patients who crossed over from crizotinib to brigatinib (HR=0.81, 95% CI, 0.53-1.22, log-rank *p*=0.331). Further updated outcome reports of RCTs need to be followed to determine the effect of each ALK-TKI on OS, as this may revise decisions with regard to the choice of first-line ALK-TKIs.

CNS metastasis is a major concern in lung cancer. CNS metastases are present at diagnosis in ∼30% of patients with *ALK*-positive NSCLC (37). First-generation crizotinib is limited in its ability to penetrate CNS and hence in most cases the disease progression site is CNS, particularly when baseline brain metastases are present [19]. A second-generation ALK-TKI such as brigatinib appears to be preferable to crizotinib for the treatment of brain metastases due to its high intracranial efficacy. The intracranial ORR was believed to be influenced by the ability to penetrate the blood-brain barrier (BBB) (38). Newly developed ALK-TKIs with improved BBB penetration such as alectinib, ceritinib, brigatinib, or lorlatinib have demonstrated significant intracranial activity that should contribute to improved overall survival. The presence of the dimethylphosphine oxide (DMPO) group in brigatinib was hypothesized to contribute to its high CNS efficacy (39). Our current study also demonstrated robust intracranial efficacy of brigatinib in treating patients with any baseline brain metastases or measurable CNS metastases (iORR=54%, median iPFS=19.26 months). In the ALTA-1L trial, the risk of intracranial progression was reduced by 56% in all patients (HR = 0.44) and by 71% in patients with any brain metastases at baseline (HR=0.29) with brigatinib compared with crizotinib (27). Brigatinib also showed superior intracranial OS versus crizotinib in patients with baseline brain metastases (HR=0.42, log-rank *P* =0.02), suggesting a survival benefit in patients with brain metastases receiving brigatinib as the first ALK-TKI treatment (27). In addition, a recent meta-analysis also compared the intracranial response of second generation of ALK inhibitors with crizotinib (40). Indicators of response in CNS were superior for alectinib and brigatinib compared with those of crizotinib. Odds of achieving intracranial response was significantly higher with these two drugs (OR=5.87, 95% CI, 3.49-9.87; *P*< 0.00001) (40). Chuang et al. also confirmed that brigatinib exerted better efficacy for PFS than crizotinib, especially among patients with baseline brain metastasis (35). In addition, brigatinib seems to show superiority in intracranial efficacy over other second-generation ALK-inhibitors. However, the network meta-analysis did not detect any significant differences in PFS between brigatinib and alectinib or lorlatinib among patients with baseline brain metastases.

Like other ALK-TKIs, patients with asymptomatic or stable CNS metastases were permitted in clinical trials. The role of brigatinib in the treatment of symptomatic CNS metastases is still not very clear. A single arm phase II study of brigatinib alone for patients with symptomatic or asymptomatic brain metastases in *ALK*-positive NSCLC is still ongoing (NCT04634110) (41). Furthermore, a case report addressed brigatinib efficacy in leptomeningeal response showing that two patients with *ALK*-positive NSCLC with leptomeningeal carcinomatosis who progressed during heavy pretreatment with crizotinib and ceritinib subsequently experienced prolonged benefit with brigatinib (42). Disclosure of these trial results might enlighten future use of brigatinib in treating patients with brain metastases.

### 4.3 Comparisons with chemotherapy

Since most patients with NSCLC have advanced disease at diagnosis, chemotherapy is the mainstay of management. In clinical practice, platinum-based regimens are the most widely used in the treatment of advanced NSCLC. It is reported that the PFS with platinum-based chemotherapy is approximately 2.1–6.9 months among advanced NSCLC patients (43). A randomized prospective study showed that patients with *ALK*-rearranged NSCLC who received chemotherapy only had a median PFS of 8.1 months and the iORR was only 27.3% (44). A recent meta-analysis confirmed that brigatinib significantly prolonged PFS in ALK inhibitor-naïve patients compared with chemotherapy (PFS for brigatinib: 24.00 months (18.40-43.20); PFS for chemotherapy: 8.1 months (5.8-11.1); HR=0.23, 95% CI 0.16-0.34) (34).

### 4.4 Safety

Although brigatinib has a good clinical therapeutic effect, its use is still limited owing to AEs. The most common AEs associated with brigatinib treatment in the current study are CPK increased, diarrhea, and nausea. A recent study has reported high incidence of any grade of CPK increased (81%) (22). It is notable that only 24% of patients had grade ≥3 CPK increased, and no cases of clinically diagnosed rhabdomyolysis were reported (27). The incidence of grade ≥3 AEs was generally low (<5%) (22, 25, 28, 29, 31), although recent ALTA-1L final results showed moderate incidence of increased CPK (26%) and lipase (15%) (27). Low to moderate rates of brigatinib discontinuation (13%, 18/136) and dose reduction (44%, 60/136) due to AEs (27), the more reliable indicators of meaningful toxicity, showed that the safety profile of brigatinib has been consistent (45).

### 4.5 Strengths and limitations

Our study has several notable limitations. First, the current meta-analysis included both clinical trials and real-world evidence studies. The variances in study design, and most importantly, the variances in baseline characteristics of study participants, might provide skewed results. For example, the definitions of PFS varied between clinical trial and real-world study. Second, the studies included had a short follow-up period, with the longest being 40.5 months and shortest being 9.3 months. Therefore, overall survival could not be investigated. Third, the current study contains a relatively small sample size, and therefore a subgroup analysis by treatment line was not feasible for each efficacy outcome. A further study with larger sample size is warranted for the disclosure of all efficacy outcome comparisons by treatment line. Fourth, brigatinib was used in different treatment lines in the included studies. Large variance of outcome efficacy was therefore reported and subgroup analysis by treatment line was not always possible. Finally, although an *in vitro* study has indicated that brigatinib is associated with a wide spectrum of *ALK* resistance mutations (19), sparse clinical reports can be found to elucidate the potential associations. More evidence is awaited to be depicted in future clinical and meta-analysis studies.

## 5 Conclusion

To summarize, brigatinib is effective in the treatment of patients with *ALK*-positive NSCLC, and it particularly yielded substantial intracranial responses and iPFS in patients with baseline brain metastases. Brigatinib used as first-line treatment yielded superior PFS compared with brigatinib used as other treatment lines. All adverse events are manageable, with gastrointestinal reactions and CPK increased found to be the most common types.

## Data availability statement

The data analyzed in this study is subject to the following licenses/restrictions: The datasets, including the redacted study protocol, redacted statistical analysis plan, and individual participants data supporting the results reported in this article, will be made available within three months from initial request, to researchers who provide a methodologically sound proposal. The data will be provided after de-identification, in compliance with applicable privacy laws, data protection and requirements for consent and anonymization. Requests to access these datasets should be directed to xingpuyuan@163.com.

## Author contributions

(I) Conceptualization: PX. and JL. (II) Methodology: XH, XZ, and JL. (III) Formal analysis: PX. (IV) Investigation: PX, XZ, and JL. (V) Data extraction: PX, XH, and XZ. (VI) Writing – Original Draft Preparation: PX and JL. (VII) Writing– Review & Editing: PX, XH, XZ and JL. (VIII) Funding Acquisition: JL. All authors contributed to the article and approved the submitted version.

## Funding

This work was supported by Takeda (China) International Trading Co., Ltd. an affiliate of Takeda Pharmaceutical Company.

## Acknowledgments

Medical writing support was provided by Fangzhou Wang, Chang Cui and Ying Wang of Happy Life Tech Co., Ltd. (HLT, an affiliate of Yidu Tech) and funded by Takeda (China) International Trading Co., Ltd. an affiliate of Takeda Pharmaceutical Company.

## Conflict of interest

Dr. Junling Li has received speaker honorarium for serving on advisory board of Takeda (China) International Trading Co., Ltd.

The remaining authors declare that the research was conducted in the absence of any commercial or financial relationships that could be construed as a potential conflict of interest. 

## Publisher’s note

All claims expressed in this article are solely those of the authors and do not necessarily represent those of their affiliated organizations, or those of the publisher, the editors and the reviewers. Any product that may be evaluated in this article, or claim that may be made by its manufacturer, is not guaranteed or endorsed by the publisher.
