# Supplementary material for: Efficacy and safety of brigatinib in ALK-positive non-small cell lung cancer treatment: A systematic review and meta-analysis
Source: Front Oncol. 2022 Nov 3;12:920709. doi: 10.3389/fonc.2022.920709 (PMC9669367; doi:10.3389/fonc.2022.920709)
Supplement: Supplementary file 1 [file DataSheet_1.pdf]

Supplementary Material

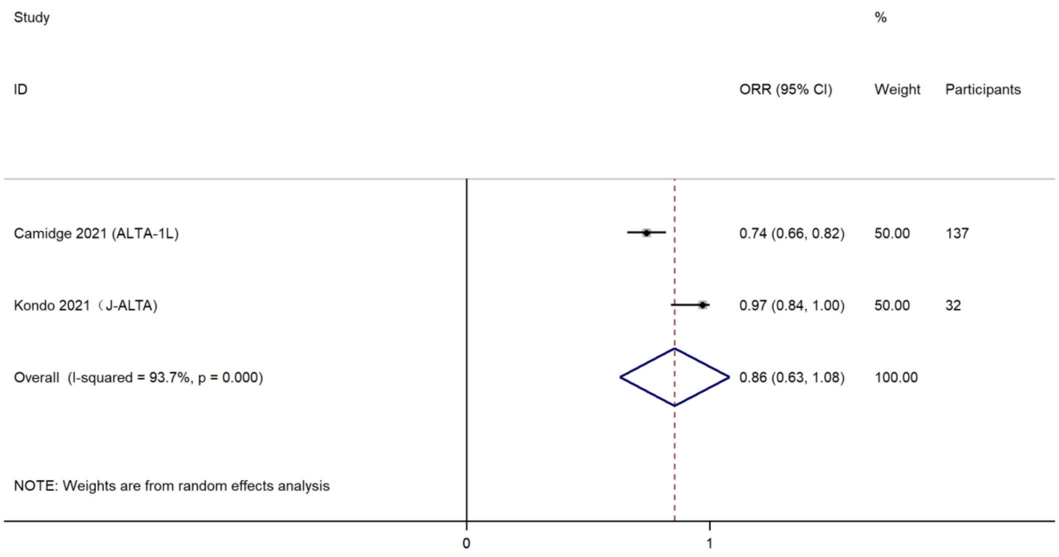

Supplemental Figure 1. Forest plot of objective response rate (ORR) among patients who received brigatinib as first-line treatment.

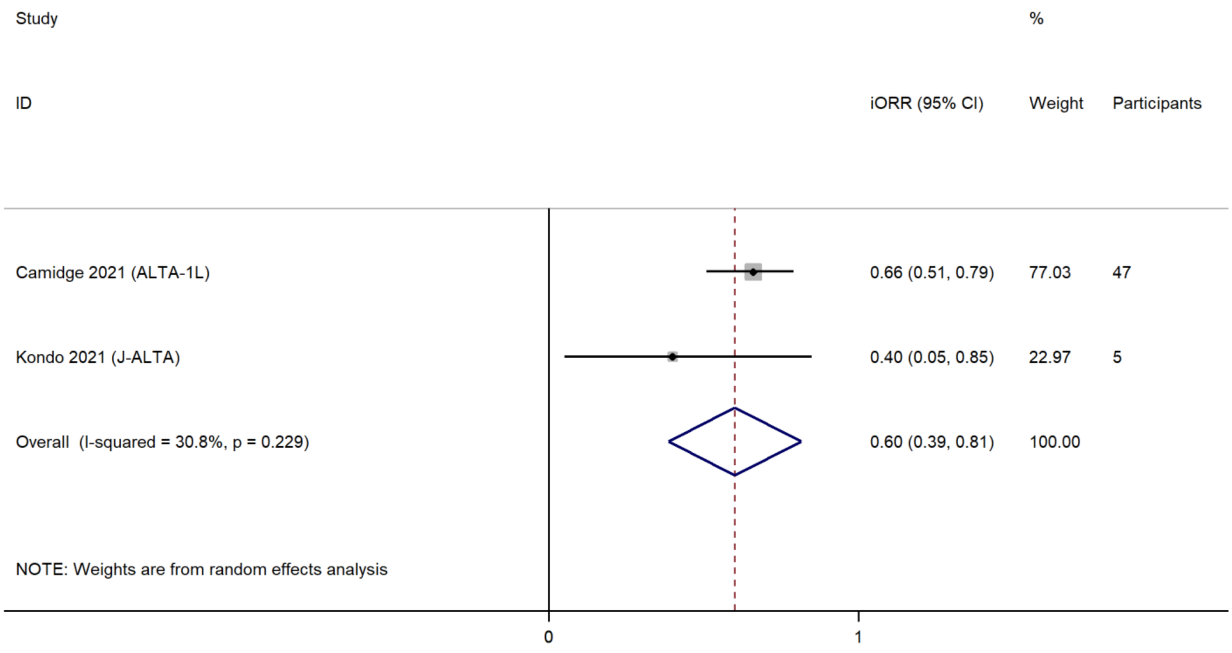

Supplemental Figure 2. Forest plot of intracranial objective response rate (iORR) among patients who received brigatinib as first-line treatment.

(3A)

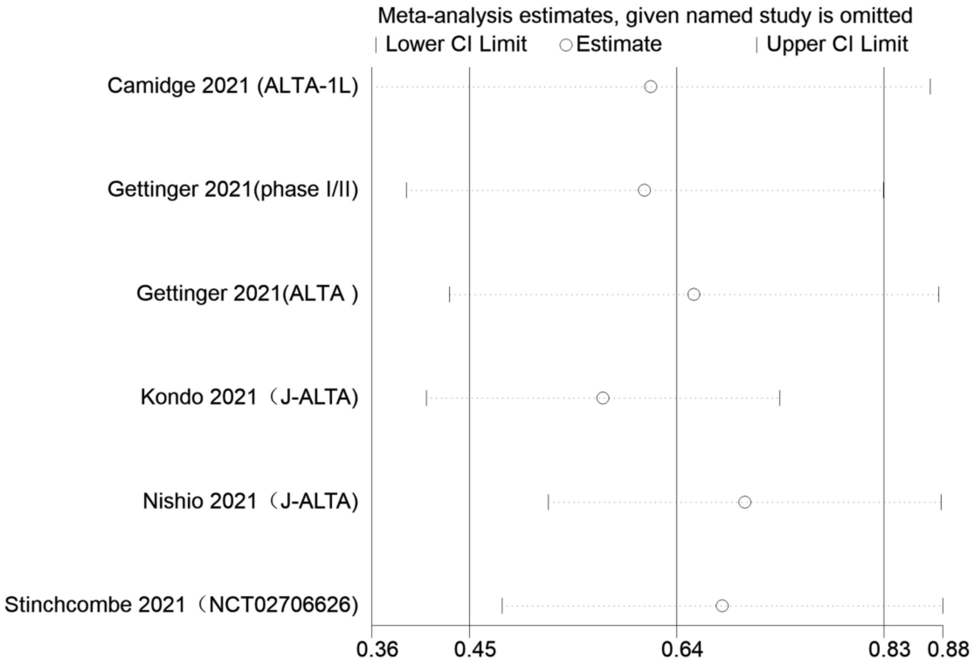
Meta-analysis estimates, given named study is omitted
Lower CI Limit
Estimate
Upper CI Limit
Camidge 2021 (ALTA-1L)
Gettinger 2021(phase I/II)
Gettinger 2021(ALTA)
Kondo 2021（J-ALTA)
Nishio 2021（J-ALTA)
Stinchcombe 2021（NCT02706626)
0.36
0.45
0.64
0.83
0.88

(3B)

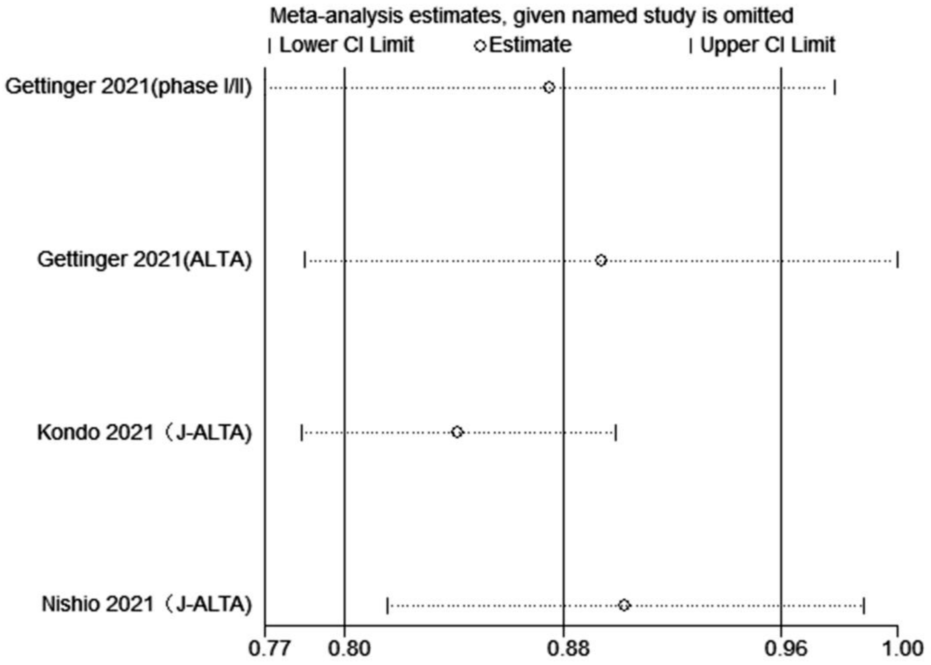
Meta-analysis estimates, given named study is omitted
Lower CI Limit
Estimate
Upper CI Limit
Gettinger 2021(phase I/II)
Gettinger 2021(ALTA)
Kondo 2021（J-ALTA)
Nishio 2021（J-ALTA)
0.77
0.80
0.88
0.96
1.00

(3C)

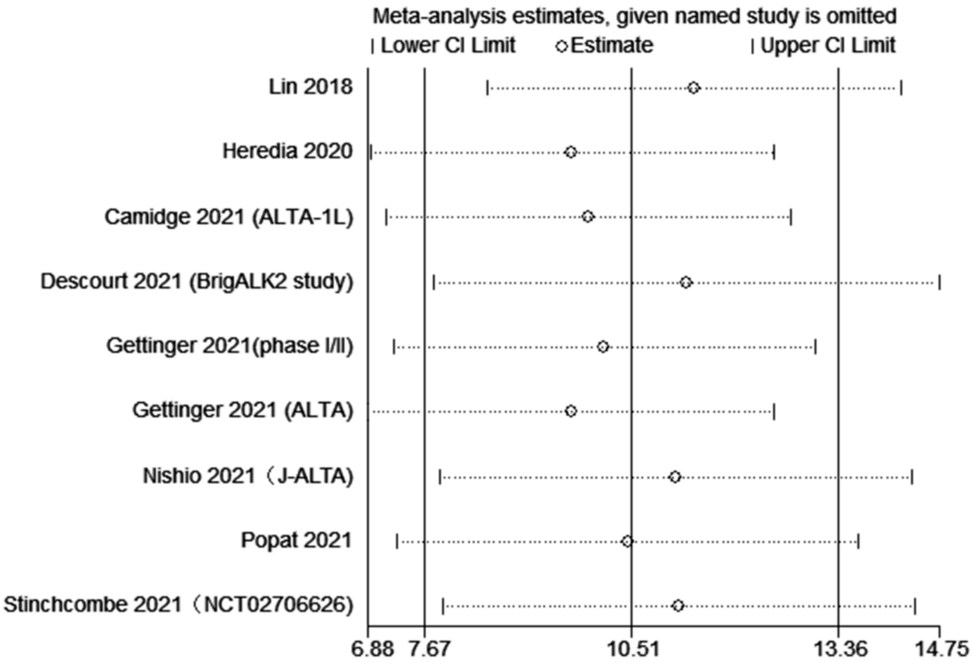

Supplemental Figure 3. A, Sensitivity analysis of objective response rate (ORR); B, Sensitivity analysis of disease control rate (DCR); C, Sensitivity analysis of progression free survival (PFS).

(4A)

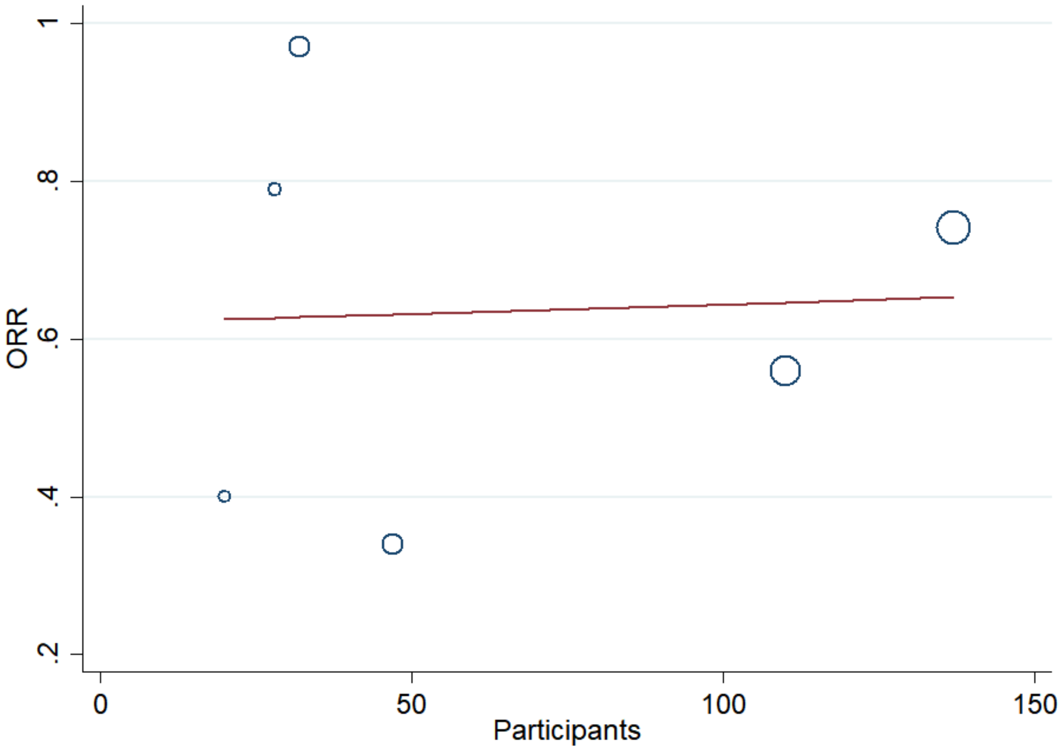


```
Meta-regression                                   Number of obs   =         6
REML estimate of between-study variance           tau2            =    .06782
% residual variation due to heterogeneity         I-squared_res   =    93.41%
Proportion of between-study variance explained    Adj R-squared   =   -28.18%
With Knapp-Hartung modification
```

orr	Coef.	Std. Err.	t	P>|t|	[95% Conf.	Interval]
participants	.0002471	.0024537	0.10	0.925	-.0065654	.0070595
_cons	.6192675	.1924284	3.22	0.032	.0850005	1.153535

(4B)

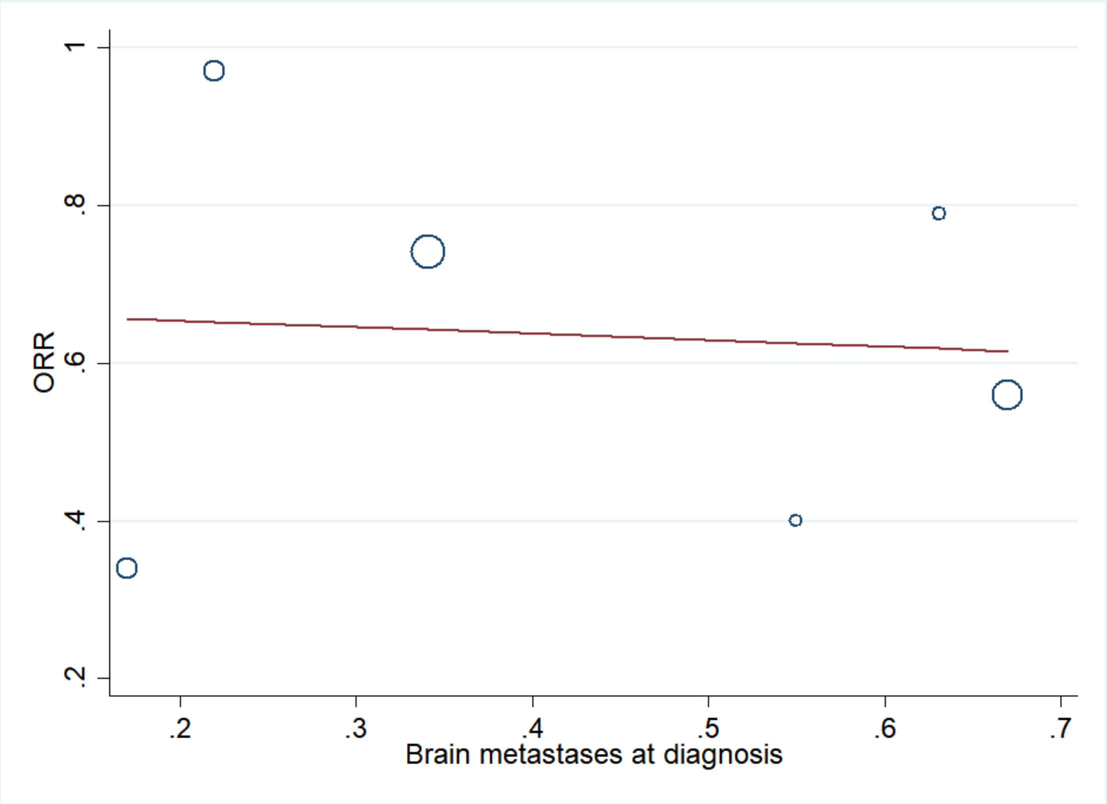

Meta-regression	Number of obs	=	6
**REML** estimate of between-study variance	tau2	=	.06733
% residual variation due to heterogeneity	I-squared_res	=	93.21%
Proportion of between-study variance explained	Adj R-squared	=	-27.24%
**With** Knapp-Hartung modification			

orr	Coef.	Std. Err.	t	P>|t|	[95% Conf.	Interval]
brainmetastasesatdiagnosis	-.0821656	.5592249	-0.15	0.890	-1.634823	1.470492
_cons	.670191	.2628434	2.55	0.063	-.0595793	1.399961

Supplemental Figure 4. A, Meta-regression analysis for heterogeneity of objective response rate (ORR) regarding sample size; B; Meta-regression analysis for heterogeneity of objective response rate (ORR) regarding brain metastases.

(5A)

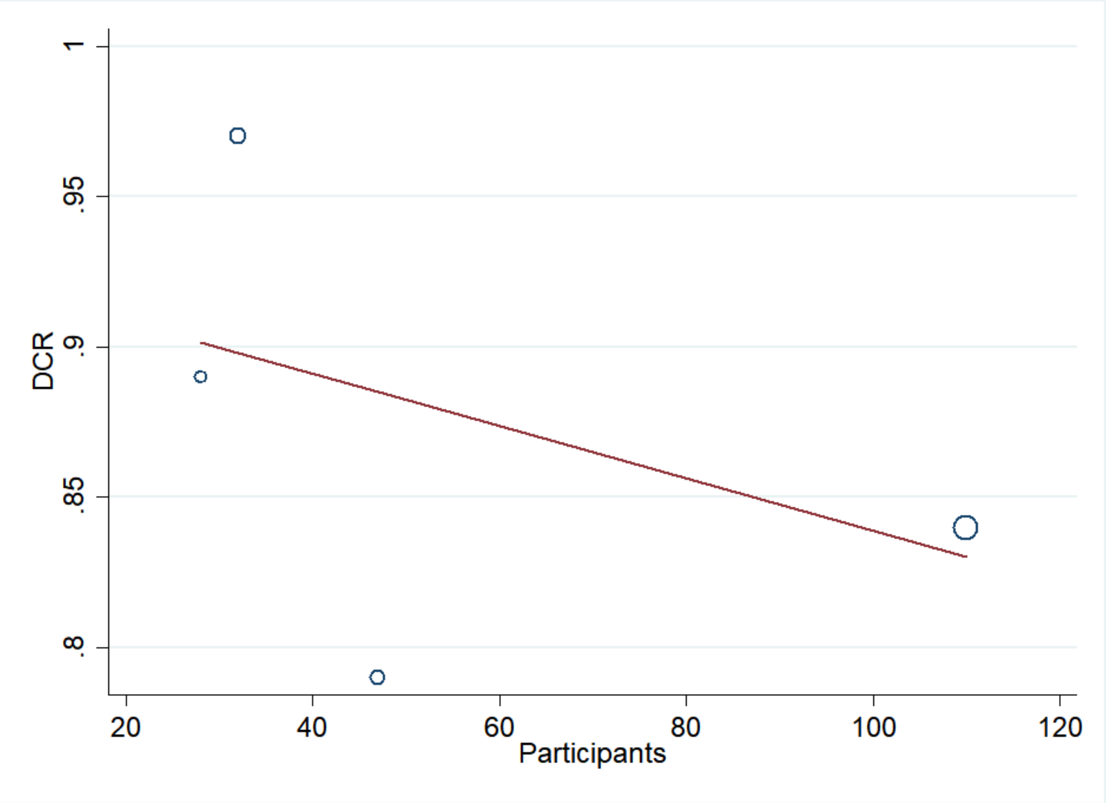


```
Meta-regression                                    Number of obs   =         4
REML estimate of between-study variance            tau2            =  .002547
% residual variation due to heterogeneity          I-squared_res   =   27.81%
Proportion of between-study variance explained     Adj R-squared   =  -96.14%
With Knapp-Hartung modification
```

dcr	Coef.	Std. Err.	t	P>|t|	[95% Conf.	Interval]
participants	-.0008715	.0011434	-0.76	0.526	-.0057913	.0040483
_cons	.9260117	.0836344	11.07	0.008	.566162	1.285861

(5B)

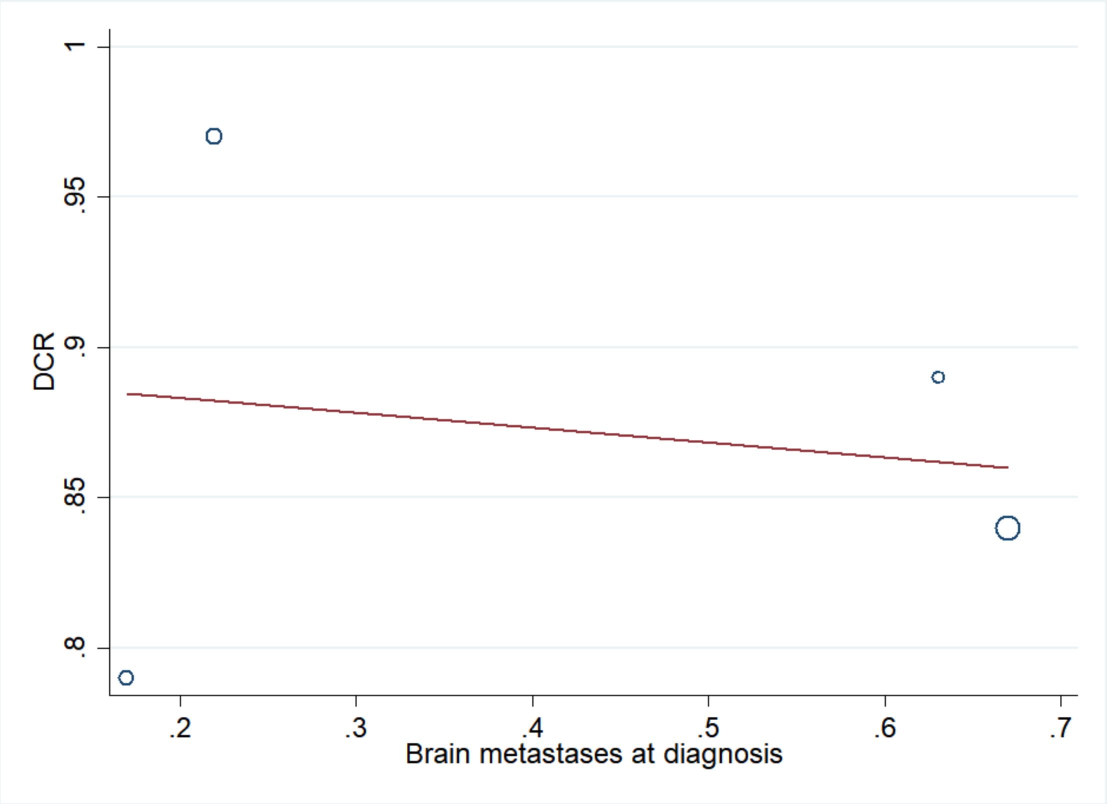


```
Meta-regression                                  Number of obs  =        4
REML estimate of between-study variance          tau2           = .003883
% residual variation due to heterogeneity        I-squared_res  =  43.41%
Proportion of between-study variance explained   Adj R-squared  = -199.04%
With Knapp-Hartung modification
```

dcr	Coef.	Std. Err.	t	P>|t|	[95% Conf.	Interval]
brainmetastasesatdiagnosis	-.0495535	.1985982	-0.25	0.826	-.9040524	.8049454
_cons	.8931612	.0989432	9.03	0.012	.4674429	1.318879

Supplemental Figure 5. A, Meta-regression analysis for heterogeneity of disease control rate (DCR) regarding sample size; B; Meta-regression analysis for heterogeneity of disease control rate (DCR) regarding brain metastases.

(6A)

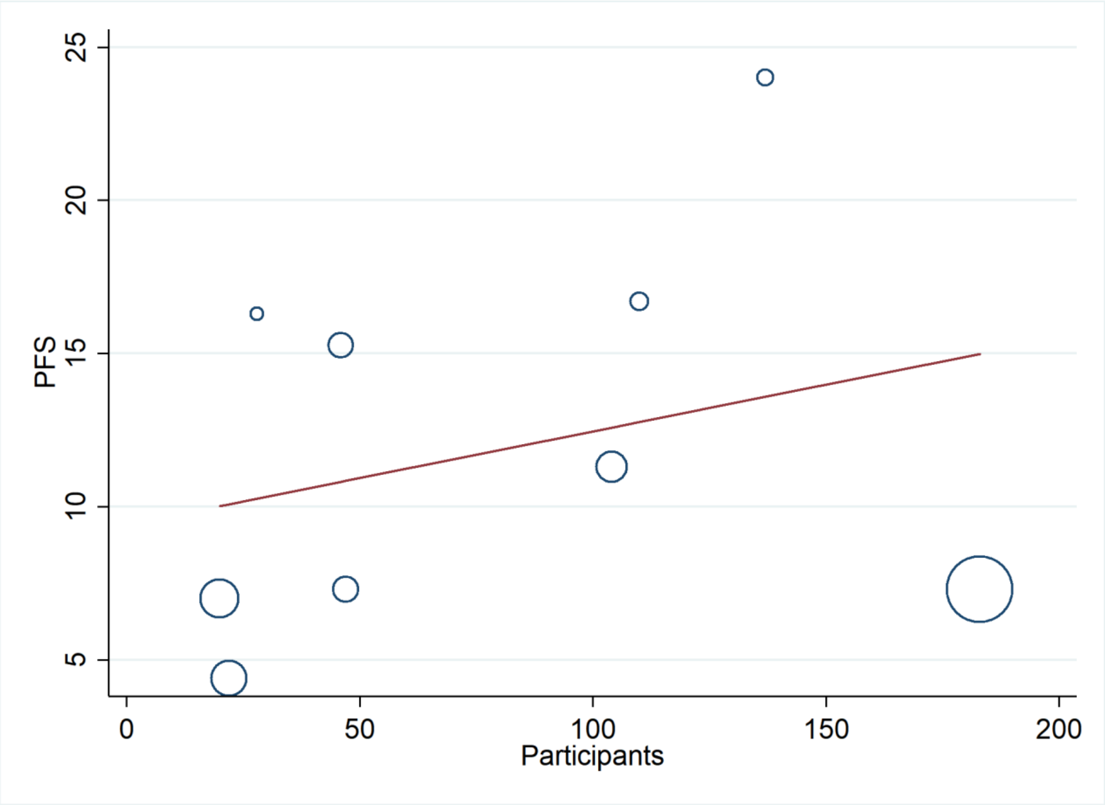


```
Meta-regression                                    Number of obs  =        9
REML estimate of between-study variance            tau2           =    36.35
% residual variation due to heterogeneity          I-squared_res  =   90.75%
Proportion of between-study variance explained     Adj R-squared  =   -7.53%
With Knapp-Hartung modification
```

pfs	Coef.	Std. Err.	t	P>|t|	[95% Conf.	Interval]
participants	.0304864	.0382823	0.80	0.452	-.0600367	.1210096
_cons	9.406849	3.669978	2.56	0.037	.7287293	18.08497

(6B)

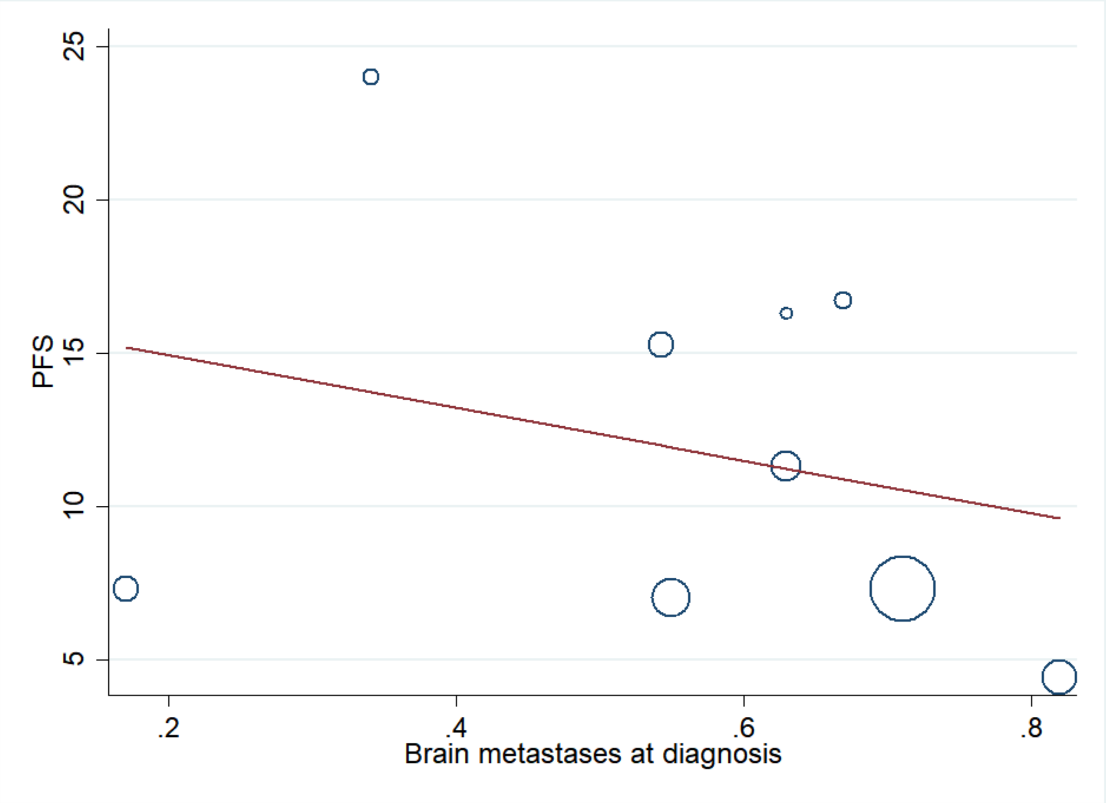


```
Meta-regression                                       Number of obs  =         9
REML estimate of between-study variance               tau2           =     36.17
% residual variation due to heterogeneity             I-squared_res  =    89.32%
Proportion of between-study variance explained        Adj R-squared  =    -7.00%
With Knapp-Hartung modification
```

pfs	Coef.	Std. Err.	t	P>|t|	[95% Conf.	Interval]
brainmetastasesatdiagnosis	-8.580177	11.43503	-0.75	0.478	-35.61973	18.45937
_cons	16.63752	6.815945	2.44	0.045	.5203719	32.75467

Supplemental Figure 6. A, Meta-regression analysis for heterogeneity of progression free survival (PFS) regarding sample size; B; Meta-regression analysis for heterogeneity of progression free survival (PFS) regarding brain metastases.

(7A)

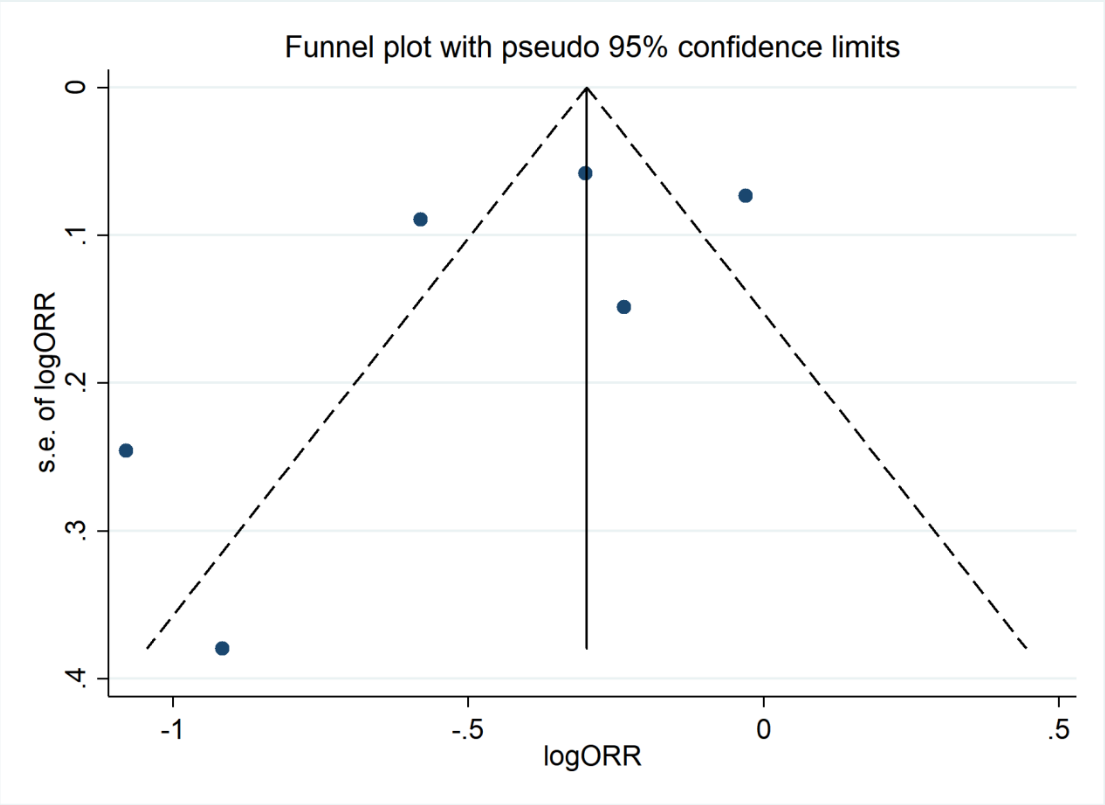
Funnel plot with pseudo 95% confidence limits
0
.1
.2
.3
.4
s.e. of logORR
-1
-.5
0
.5
logORR

(7B)

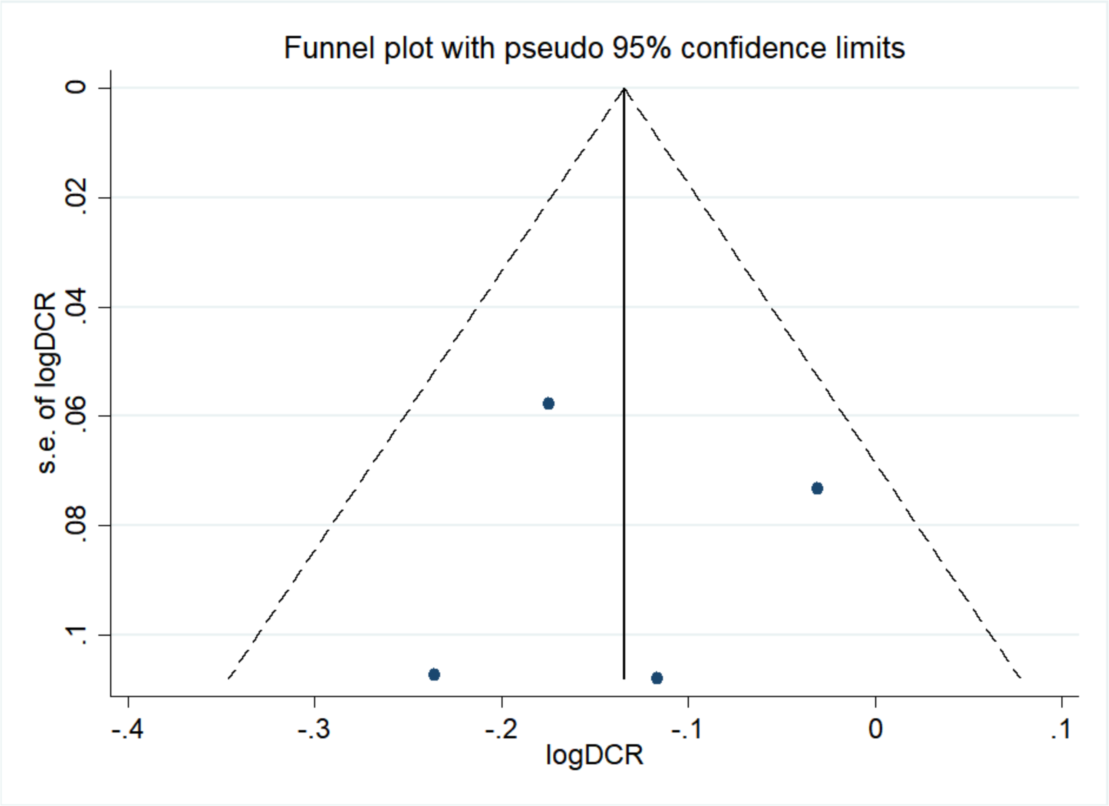
Funnel plot with pseudo 95% confidence limits
s.e. of logDCR
0
.02
.04
.06
.08
.1
-.4
-.3
-.2
-.1
0
.1
logDCR

(7C)

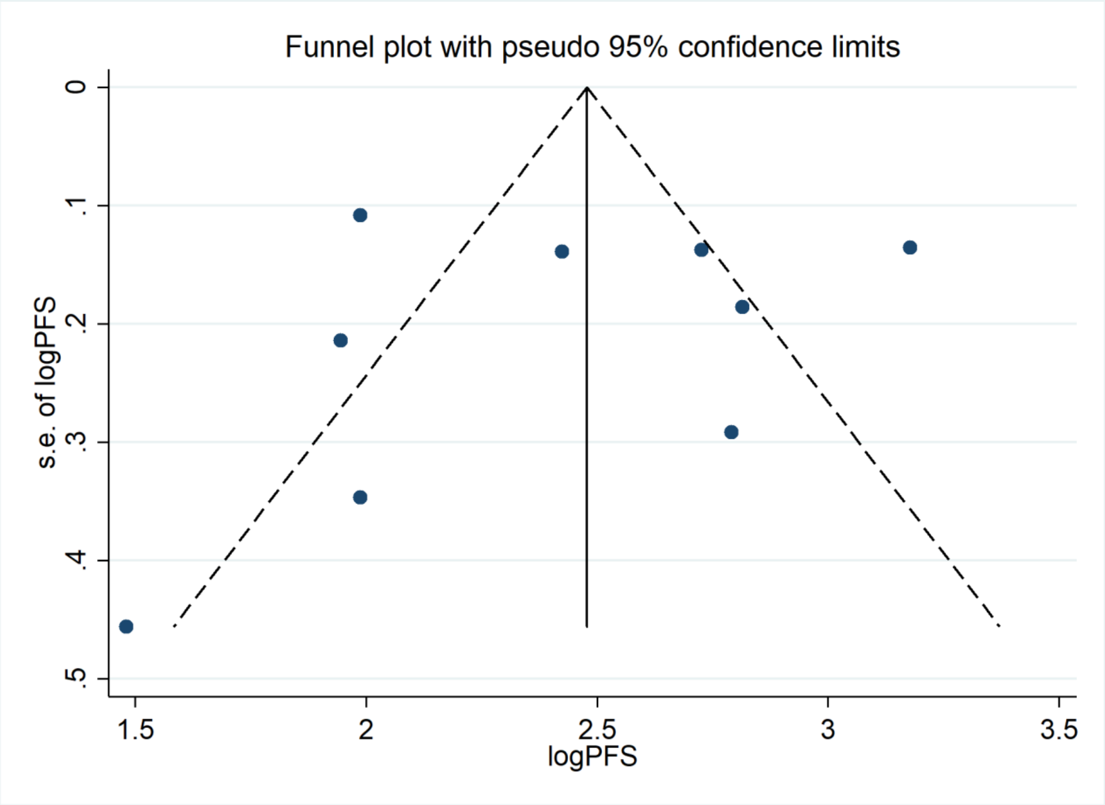
Funnel plot with pseudo 95% confidence limits
s.e. of logPFS
0
.1
.2
.3
.4
.5
1.5
2
2.5
3
3.5
logPFS

(7D)

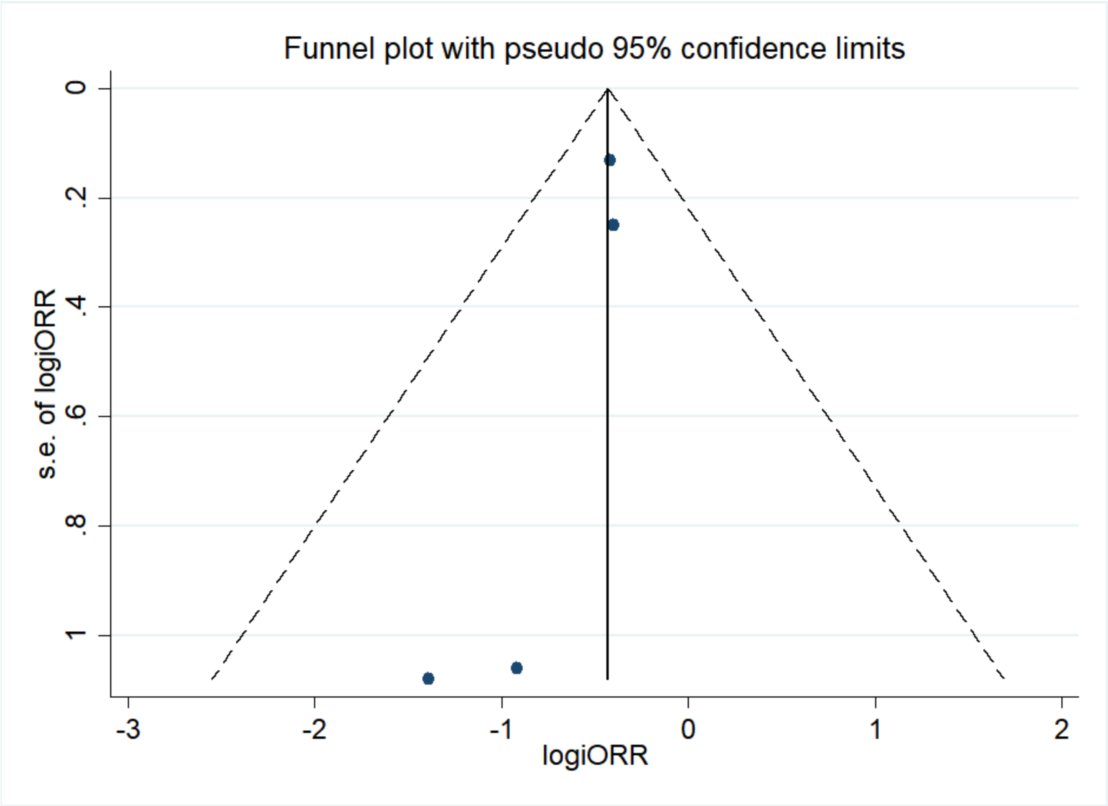

(7E)

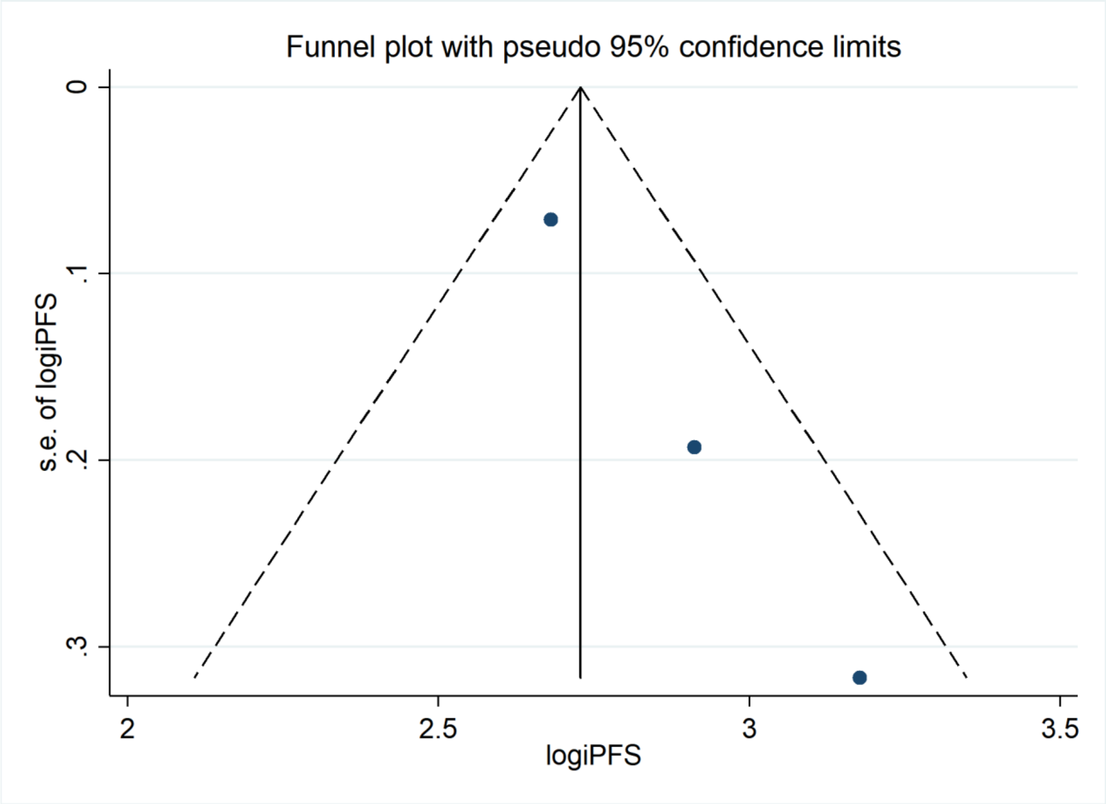

Supplemental Figure 7. A, Funnel plot of objective response rate (ORR); B, Funnel plot of disease

control rate (DCR); C, Funnel plot of progression free survival (PFS); D, Funnel plot of intracranial objective response rate (iORR); E, Funnel plot of intracranial progression free survival (iPFS).

Supplemental Table 1. Treatment line and number of patients for included studies.

Study	Treatment line	Number of patients
Camidge 2018 (phase I/II)	1L, 2L post-crizotinib	46
Lin 2018	2L post-alectinib	22
Heredia 2020	≥2L	46
Descourt 2021 (BrigALK2 study)	≥2L	183
Camidge 2021 (ALTA-1L)	1L	137
Nishio 2021 (J-ALTA)	≥2L post alectinib±crizotinib	47
Stinchcombe 2021 (NCT02706626)	≥2L	20
Popat 2021	≥2L	104
Gettinger 2021 (phase I/II)	1L, 2L post-crizotinib	28 (1L, n=3; 2L post-crizotinib, n=25)
Gettinger 2021 (ALTA)	2L post-crizotinib	110
Kondo 2021 (J-ALTA)	1L	32

Supplemental Table 2. Publication bias based on Begg's test and Egger's test

Comparisons	Begg's test		Egger's test	
	Z	***P***	***t***	***P***
ORR	0.38	0.707	-1.27	0.273
iORR	1.70	0.089	-2.64	0.118
PFS	0.94	0.348	-0.31	0.763
iPFS	1.04	0.296	29.46	0.022
DCR	-0.34	1.000	-0.09	0.938
