# Supplementary material for: Efficacy and safety of brigatinib in ALK-positive non-small cell lung cancer treatment: A systematic review and meta-analysis
Source: Front Oncol. 2022 Nov 3;12:920709. doi: 10.3389/fonc.2022.920709 (PMC9669367; doi:10.3389/fonc.2022.920709)
Supplement: Supplementary file 2 [file Presentation_1.pdf]

Appendix 1. Quality assessment

CASP checklist for cohort study

1. Did the study address a clearly focused on issue?
2. Was the cohort recruited in an acceptable way?
3. Was the exposure accurately measured to minimize bias?
4. Was the outcome accurately measured to minimize bias?
5. (a)Have the authors identified all important confounding factors?
   (b) Have they taken account of the confounding factors in the design and/or analysis?
6. (a)Was the follow up of subjects complete enough?
   (b)Was the follow up of subjects long enough?
7. What are the results of this study?
8. How precise are the results?
9. Do you believe the results?
10. Can the results be applied to the local population?
11. Do the results of this study fit with other available evidence?
12. What are the implications of this study for practice?

Article 1

Exploratory Analysis of Brigatinib Activity in Patients With Anaplastic Lymphoma Kinase-Positive Non–Small-Cell Lung Cancer and Brain Metastases in Two Clinical Trials

Question 1 Yes.

In patients with crizotinib-treated, anaplastic lymphoma kinase gene (ALK)-rearranged non–small-cell lung cancer (*ALK*-positive NSCLC), initial disease progression often occurs in the CNS. We evaluated brigatinib, a next-generation ALK inhibitor, in patients with *ALK*-positive NSCLC with brain metastases.

Question 2 Yes.

Trials were conducted in accordance with the Declaration of Helsinki and International Council for Harmonisation guidelines for good clinical practice. All patients provided written informed consent.

Question 3 Yes.

In both studies, disease assessment (per RECIST v1.1) included imaging of the chest and abdomen at screening and every 8 weeks until progression. Contrast-enhanced brain magnetic resonance imaging (MRI) was required at screening in all patients and performed every 8 weeks in patients with investigator-assessed baseline brain metastases. In ALTA, after cycle 15, disease was assessed every 12 weeks until progression. Objective responses were confirmed $ 4 weeks post response. In phI/II and ALTA, MRI scans were analyzed by neuroradiologists in the respective IRCs. Reviewers were blinded to investigator assessment and systemic response. The IRCs assessed screening MRI scans in all patients. Up to five measurable brain metastases were chosen as target lesions. In phI/II, target lesions could have been previously irradiated. In ALTA, brain lesions were not used as target lesions if they were previously treated with whole-brain radiation therapy (within 3 months), stereotactic radiosurgery, or surgical resection, unless there was un ambiguous radiologic progression after radiotherapy. Intracranial response in patients with one or more measurable brain lesions was defined using criteria based on RECIST: $ 30% decrease in the sum of the longest diameters of target lesions and non-progression in nontarget lesions. Only complete responses could be recorded in patients with non-measurable brain metastases, for whom response was defined as the disappearance on all brain lesions.

Question 4 Yes.

PhI/II efficacy analyses were performed in evaluable patients (i.e., those with one or more on-study scans), whereas ALTA efficacy analyses were performed in the subset of the intention-to-treat population with baseline brain metastases, regardless of on-study scans. The exact binomial method was used to calculate 95% CIs for

proportions. Kaplan-Meier analysis was used to estimate median values and two-sided 95% CIs for time-to-event data (duration of response, PFS, and overall survival). Investigator-assessed data are reported as of May 31, 2016, for both trials. IRC-assessed data had last scan dates of October 8, 2015, for phI/II and July 13, 2016, for ALTA. The ALTA trial was not designed for statistical comparisons between arms; however, post hoc hazard ratios were estimated for time-to-event analyses (e.g., PFS, overall survival) to support selection of the recommended dose. Statistical analyses were performed using SAS software (version 9.4).

Question 5(a)   Can't tell.

Question 5(b)   Can't tell.

Question 6(a)   Yes.

Question 6(b)   Yes.

Question 7   The numerical value of iPFS.

Question 8   The median iPFS on brigatinib was 15.6 months (95% CI: 9.0–18.3months).

Question 9   Can't tell.

The sample size isn't enough.

Question 10   Yes.

Patients were identified at three participating in situations: Massachusetts General Hospital (MGH; n =11), Memorial Sloan Kettering Cancer Center (n=6), and University of California–Irvine (n =5). All patients had advanced NSCLC with an ALK rearrangement identified by local molecular profiling.

Question 11   Can't tell.

There is no available evidence to the result.

Question 12   It can provide evidence to solve the question that whether Brigatinib is available to *ALK*-Positive NSCLC.

Article 2
Brigatinib in Patients With Alectinib-Refractory *ALK*-Positive NSCLC

Question 1　Yes.
Here, based on a multicenter, retrospective analysis of 22 *ALK*-positive patients treated with alectinib followed by brigatinib, we report the efficacy and safety of brigatinib in the setting of alectinib resistance.

Question 2　Yes.
This study was approved by the Institutional Review Board at each participating institution.

Question 3　Yes.
Medical records were retrospectively reviewed, and data were extracted on clinical, pathologic, and molecular features as well as treatment histories. Overall and intracranial responses to therapy were determined using the Response Evaluation Criteria in Solid Tumors version 1.1 based on investigator assessment.

Question 4　Yes.
PFS was measured from the time of brigatinib or alectinib treatment initiation to clinical/radiographic progression or death. Patients without documented disease progression were censored on the date of last follow-up. Duration of treatment was measured from the time of brigatinib or alectinib initiation to the date that the drug was dis continued, or — if continuing on brigatinib at the time of data analysis — censored on the date of last follow-up. All data were updated as of April 15, 2018.
PFS and duration of treatment endpoints were estimated using the Kaplan-Meier method. 95% confidence intervals (CIs) were calculated using the log-log trans formation. Data analysis was performed using SAS 9.4(SAS Institute, Cary North Carolina).

Question 5(a)　Can't tell.
Question 5(b)　Can't tell.

Question 6(a)　Yes.
PFS was measured from the time of brigatinib or alectinib treatment initiation to clinical/radiographic progression or death. Patients without documented disease progression were censored on the date of last follow-up. Duration of treatment was measured from the time of brigatinib or alectinib initiation to the date that the drug was dis continued, or — if continuing on brigatinib at the time to data analysis — censored on the date of last follow-up. All data were updated as of April 15, 2018.

Question 6(b)　Yes.

The median PFS on brigatinib was 4.4 months (95% CI: 1.8–5.6 months). The median PFS can be identified.

Question 7 The numerical value of PFS.

Question 8 The median PFS on brigatinib was 4.4 months (95% CI: 1.8–5.6 months).

Question 9 Can't tell.

The sample size isn't enough.

Question 10 Yes.

Patients were identified at three participating in situations: Massachusetts General Hospital (MGH; n =11), Memorial Sloan Kettering Cancer Center (n=6), and University of California–Irvine (n =5). All patients had advanced NSCLC with an ALK rearrangement identified by local molecular profiling.

Question 11 Can't tell.

There is no available evidence to the result.

Question 12 It can provide evidence to solve the question that whether Brigatinib is available to *ALK*-Positive NSCLC.

Article 3

BrigALK2 study: a multicentric real-world study evaluating brigatinib in ALK positive advanced pretreated non-small-cell lung cancers: long-term follow-up, with focus on lorlatinib efficacy after brigatinib

Question 1 Yes.

This retrospective multicentric study analyzed *ALK*-positive advanced NSCLC patients pretreated with at least one tyrosine-kinase inhibitor, including crizotinib, and enrolled in the brigatinib French early access program.

Question 2 Yes.

The study was conducted in accordance with the Declaration of Helsinki and was approved by

the French Advisory Committee on Information Processing in Health Research (CCTIRS).

Question 3 Yes.

Patient data were obtained retrospectively from medical files and included demographics, characteristics of NSCLC, number and localization of metastatic sites, previous treatments, tumor response to brigatinib, resistance mutation before brigatinib initiation or after progression and treatments after progression. Patients were included consecutively in each center according to inclusion criteria without selection.

Question 4 Yes.

The Kaplan-Meier method was used to estimate PFS and OS for the entire cohort and in defined

subgroups according to the number of lines of treatments. Best response to treatment was assessed according to RECIST 1.1 criteria [16]. The statistical analyses were performed with SAS 9.4 software (SAS Institute, Cary, NC, USA).

Question 5(a) Can't tell.
Question 5(b) Can't tell.

Question 6(a) Yes.(?)
Question 6(b) No.

The median PFS can be identified. But the OS can't.

Question 7 The numerical value of PFS and OS.

Question 8 The date of primary data cut-off was June 30, 2018. Median PFS from initiation of brigatinib was 6.6 (95% CI, 4.8–9.9) months with a median OS

of 17.2 (95% CI, 11.0–not reached) months.

Question 9 Yes.

Question 10 Yes.

The inclusion criteria were the followings: at least 18 years old; advanced NSCLC; ALK positive NSCLC assessed with in situ hybridization (FISH) and/or immunohistochemistry; previous treatment with at least one ALK inhibitor including crizotinib; treatment with brigatinib in the setting of the early access program from September 1st, 2016 to January 1st, 2018.

Question 11 Can't tell.

There is no available evidence to the result.

Question 12 It can provide evidence to solve the question that whether Brigatinib is available to *ALK*-Positive NSCLC.

Article 4

Brigatinib versus Crizotinib in Anaplastic Lymphoma Kinase (ALK) Inhibitor–Naive Advanced *ALK*-Positive Non–Small Cell Lung Cancer: Final Results of the Phase 3 ALTA-1L Trial

Question 1 Yes.

This study reports the results of the second interim analysis (150 events) of the phase III ALTA-1L study of first-line treatment with brigatinib versus crizotinib in patients with anaplastic lymphoma kinase–positive (ALK1) non–small cell lung cancer(NSCLC).

Question 2 Yes.

All patients provided written informed consent. Protocol and consent documents were approved by local in situational review boards or ethics committees. The trial was conducted in accordance with the ethical standards of the Declaration of Helsinki and International Council for Harmonization guidelines for good clinical practice.

Question 3 Yes.

Chest and abdomen (computed tomography or magnetic resonance imaging [MRI] with contrast) and brain (MRI with contrast) imaging was performed at screening, every 8 weeks through cycle 14 (28 d/cycle), and then every 12 weeks through treatment discontinuation. Two BIRCs

performed disease assessments: one evaluated all disease on the basis of RECIST version 1.1,25 and one evaluated intracranial CNS disease. Confirmation of response occurred $ 4 weeks after initial response. Adverse events (AEs) were categorized according to National Cancer Institute Common Terminology Criteria for AEs, version 4.03. Patients completed the validated European Organization for Research and Treatment of Cancer (EORTC) QoL Questionnaire (QLQ)-C30 (version 3.0)26 and its lung cancer–specific module (QLQ-LC13 version 3.0)27 at baseline, day 1 of every 4-week cycle until end of treatment, end of treatment, and 30 days after last dose.

Question 4 Yes.

The primary end point was compared between arms using a 2-sided stratified log-rank test. Time-to-event efficacy analyses estimated median values and 2-sided 95% CIs using Kaplan-Meier methods. To adjust for potential time-dependent confounding effects of crossover after patients discontinue crizotinib, an additional OS sensitivity analysis was conducted using marginal structural models (MSMs).

Question 5(a) Yes.

Question 5(b) Yes.

Question 6(a) Yes.
Question 6(b) No.
The median PFS can't be reached.

Question 7 The numerical value of PFS, ORR and iORR.
Question 8 Median PFS(95% CI) 24.0 (18.5 to NR)months
ORR (95% CI) 74% (66%,81%)
iORR(95% CI) 78% (52%,94%)

Question 9 Yes.

Question 10 Yes.
enrolled patients were adults with locally advanced/metastatic NSCLC and $ 1 measurable lesion per RECIST version 1.1 who had not received prior ALK-targeted therapy (Data Supplement). Asymptomatic or stable CNS metastases (defined as neurologically stable, without increasing doses of corticosteroids or anticonvulsant use for 7 days before randomization) were permitted.

Question 11 Can't tell.
There is no available evidence to the result.

Question 12 It can provide evidence to solve the question that whether Brigatinib is available to *ALK*-Positive NSCLC.

Article 5

Brigatinib in *ALK*-positive non-small cell lung cancer: real-world data in the Latin American population (Bri-world extend CLICaP)

Question 1 Yes.

This retrospective observational study examined the effectiveness of brigatinib in a real-world population treated in four Latin American institutions from January 2018 to March 2020.

Question 2 Yes.

This study was performed in accordance with the Declaration of Helsinki and the principles of Good Clinical Practice.

Question 3 Yes.

Question 4 Yes.

TTD, PFS and OS were estimated by the Kaplan-Meier method. All statistical tests were two-sided, and $p < 0.05$ was deemed to be statistically significant. SPSS software (version 25) was used for data analysis.

Question 5(a) Yes.

Question 5(b) Yes.

Reasons for brigatinib discontinuation were summarized as frequencies and percentages. Safety data, toxicities and drug dosage reductions were reported as frequency counts and percentages. Subgroup analyses were performed by stratifying patients considering the number of previous lines, immediate prior ALK-TKI (crizotinib, ceritinib or alectinib) therapy, CNS metastases at diagnosis and prior systemic treatment with chemotherapy.

Question 6(a) Yes.

Question 6(b) No.

The OS can't be identified.

Question 7 The numerical value of PFS.

Question 8 the median PFS was 15.2 months (95% CI: 11.6–18.8)

Question 9 Yes.

Question 10 Yes.

Inclusion criteria were patients ≥18 years of age with a pathologically confirmed

diagnosis of locally advanced or metastatic disease (stage IIIB–IV) NSCLC, LK positive according to local standard procedures [26,27] and progression after at least one prior ALK-TKI therapy or treatment discontinuation due to intolerable toxicity. Mutational status (ALKr) was confirmed in tissue biopsies by immunochemistry (D5F3) or FISH following diagnoses.

Question 11 Can't tell.

There is no available evidence to the result.

Question 12 It can provide evidence to solve the question that whether Brigatinib is available to *ALK*-Positive NSCLC.

Article 6

Brigatinib in Japanese Patients With ALK–Positive Non–Small Cell Lung Cancer Previously Treated With Alectinib and Other Tyrosine Kinase Inhibitors: Outcomes of the Phase 2 J-ALTA Trial

Question 1 Yes.

This was a single-arm, multicenter, phase 2, open-label study in Japanese patients with advanced ALK+ NSCLC (ClinicalTrials.gov identifier: NCT03410108) consisting of a safety lead-in stage followed by an expansion stage with two cohorts of ALK TKI–refractory patients and one cohort of treatment-naive patients.

Question 2 Yes.

The informed-consent and protocol documents were approved by the local institutional review board or ethics committee at each site.

Question 3 Yes.

Patients diagnosed as ALK positive by a different test could have been enrolled if adequate tissue was available for confirmation by Vysis ALK Break Apart FISH. Central confirmation of ALK rearrangement was not required before enrollment. Patients were also required to have: at least one measurable lesion by investigator assessment according to the Response Evaluation Criteria in Solid Tumors (RECIST), version 1.121; recovered from toxicities related to prior anticancer therapy; Eastern Cooperative Oncology Group (ECOG) performance status of 2 or lower, and had at least 7 days washout period between the prior TKI and the study drug brigatinib. Patients were excluded if they had previously received more than one regimen (more than three regimens for the safety lead-in) of systemic anticancer therapy (other than ALK TKIs) for locally advanced or metastatic disease; ; had a history or presence of interstitial lung disease (ILD); had current spinal cord compression; or had symptomatic CNS metastases or asymptomatic CNS metastases requiring an increasing dose of corticosteroids. Patients with asymptomatic leptomeningeal disease without cord compression were allowed.

Question 4 Yes.

The point estimate of confirmed ORR at primary analysis was calculated by the method suggested by Kunzmann22 with weight function of uniform distribution of [0,1]. Statistical inference was performed at a one-sided 0.025 level of significance or a two-sided 0.05 level of significance, as appropriate, to preserve a one-sided overall type I error rate at or below 0.025 or two-sided overall type I error rate at or below 0.05. Statistical analyses were performed using SAS version 9.4 (SAS Institute, Cary, NC).

Question 5(a) Can’t tell.

Question 5(b) Can't tell.

Question 6(a) Yes.
Question 6(b) Yes.

Question 7 The numerical value of PFS, DCR, ORR and iORR.

Question 8 IRC-assessed objective response (confirmed ORR: 34%; 95% CI, 21% – 49%)
Median IRC-assessed PFS was 7.3 months (95% CI, 3.7–9.3 months)
The confirmed iORR was 25% (95% CI, 3%–65%)
Disease control rate was 79% (95% CI, 64%–89%)

Question 9 Yes.

Question 10 Yes.
Eligible patients (≥20 years of age) had histologically or cytologically confirmed stage IIIB, stage IIIC (locally advanced or recurrent and not a candidate for definitive multimodality therapy), or stage IV NSCLC with documented ALK rearrangement. ALK rearrangement must have been documented by the Vysis ALK Break Apart fluorescence in situ hybridization (FISH) Probe Kit, the Nichirei Histofine ALK intercalated antibody-enhanced polymer (iAEP) Kit, or the Ventana ALK (D5F3) CDx Assay at any time during prior disease course.

Question 11 Can't tell.
There is no available evidence to the result.

Question 12 It can provide evidence to solve the question that whether Brigatinib is available to *ALK*-Positive NSCLC.

Article 7

Brief report: Preliminary clinical and molecular analysis results from a single arm phase 2 trial of brigatinib in patients with disease progression after next-generation anaplastic lymphoma kinase (ALK) tyrosine kinase inhibitors in advanced *ALK* + non small cell lung cancer

Question 1 Yes.

This was a single-arm, multicenter, phase 2, open-label study in Japanese patients with advanced ALK+ NSCLC (ClinicalTrials.gov identifier: NCT03410108) consisting of a safety lead-in stage followed by an expansion stage with two cohorts of ALK TKI–refractory patients and one cohort of treatment-naive patients.

Question 2 Yes.

The informed-consent and protocol documents were approved by the local institutional review board or ethics committee at each site.

Question 3 Yes.

Tumor samples were analyzed using gene fusion and gene mutation NGS assays performed in the

Colorado Molecular Correlates Laboratory in the Department of Pathology at the University of Colorado – Anschutz Medical Campus. Total nucleic acid was extracted from FFPE processed material via the Agencourt FormaPure Kit (Beckman Coulter, Brea, CA), then processed for gene fusion analysis via the Archer FusionPlex Solid Tumor library preparation kit and for gene mutation analysis by a customized version of the Archer Variant Plex Solid Tumor library preparation kit (ArcherDx, Boulder, CO). The resulting libraries were sequenced on either the Illumina MiSeq or Illumina NextSeq instruments (Illumina, San Diego, CA). Raw sequence data was analyzed using the ArcherDx Analysis software package (version 4.1.1.7 for fusions, version 5.1.2 for mutations, ArcherDx). Trained personnel manually inspected bioinformatically identified fusions and mutations.

Question 4 Yes.

The primary endpoint was objective response rate (ORR), and secondary endpoints were progression free survival (PFS), safety, and overall survival (OS). A Simon 2-stage design was used: in stage I if ≥ 2 responses were observed among 20 eligible patients, the study would proceed to stage 2 and enroll an additional 20 patients. If ≥ 5 responses were observed among 40 eligible patients then treatment would be considered worthy of further investigation. The sample size of 40 patients provided 89% power to reject the null hypothesis that the ORR is ≤ 5% when the true ORR is ≥ 20% at a one-sided significance level of 0.05. The statistical analyses were performed on SAS 9.4 (SAS Inc., Cary, NC) on data set locked on June 6, 2020. Patient data were reviewed by the study chair and the data management and statistical analyses

were provided by statisticians at Duke Cancer Institute.

Question 5(a) Can't tell.
Question 5(b) Can't tell.

Question 6(a) Yes.
Question 6(b) Yes.

Question 7 The numerical value of PFS and ORR.

Question 8 The investigator-assessed confirmed ORR was 40% (95% CI: 19% to 62%)
The median PFS was 7.0 months (95% CI: 4.6 to 10.1)
Question 9 Yes.

Question 10 Yes.
Patients were required to have advanced ALK + (based on standard of care testing) NSCLC, progression on a next generation ALK TKI, ECOG performance status of 0-2, adequate organ function, and measurable disease by RECIST 1.1.(9) There was no restriction on the number of prior therapies. Core biopsy after progression on the most recent therapy within 60 days of day 1 of study therapy was required, unless disease sites were inaccessible or biopsy posed excessive risk. All patients underwent brain imaging with a magnetic resonance imaging or computed tomography at baseline.

Question 11 Can't tell.
There is no available evidence to the result.

Question 12 It can provide evidence to solve the question that whether Brigatinib is available to *ALK*-Positive NSCLC.

Article 8
Real-world treatment outcomes with brigatinib in patients with pretreated ALK+ metastatic non-small cell lung cancer

Question 1 Yes.
UVEA-Brig was a retrospective chart review, with no comparator, designed to collect clinical data reflective of patients with ALK+ locally advanced and/or metastatic NSCLC who had received at least one prior ALK TKI and were subsequently treated with brigatinib.

Question 2 Yes.
The study was conducted in accordance with the protocol, the current version of the Declaration of Helsinki International Conference on Harmonisation E6 Good Clinical Practice: Consolidated Guideline, Good Pharmacoepidemiology Practice (GPP), International Society for Pharmacoepidemiology (ISPE) GPP guidelines, and all applicable laws and regulations. Signed informed consent forms were required in Italy, Spain, and Norway if patients were living, but were not mandatory in the UK. Signed informed consent forms were not required for medical chart data collection for deceased patients except in Norway where signed consent from the patient's parent or legal guardian was required by some Ethics Committees.

Question 3 Yes.
Data were extracted retrospectively from patient medical records using electronic case report forms.

Question 4 Yes.
The UVEA-Brig study was observational and epidemiological methods were applied for data analyses, which were performed using the SAS software. Descriptive statistics were used to present the patient characteristics and treatment patterns. OS, PFS, DoR, TTD, and DoT were determined using Kaplan-Meier analysis for each line of brigatinib therapy. Complete response (CR), partial response (PR), stable disease (SD), and progressive disease (PD) rates were calculated as proportions by line of brigatinib therapy initiation. Response to therapy was defined as tumor shrinkage or disappearance, where possible assessed using RECIST v1.1.

Question 5(a) Can't tell.
Question 5(b) Can't tell.

Question 6(a) Yes.
Question 6(b) No.

Question 7 The numerical value of PFS.

Question 8 median PFS was 11.3 (95% CI:8.6–12.9) months
Question 9 Yes.

Question 10 Yes.
As of December 2017, 352 patients had been included in the EAP at 98 sites in nine European countries (Austria, France, Germany, Ireland, Italy, Norway, Spain, Switzerland and the UK). Sites eligible for inclusion in the UVEA-Brig study were those at which at least two patients were treated with brigatinib in the EAP, local rules and regulations allowed data capture of EAP patients, and no other competing study was going on.

Question 11 Can't tell.
There is no available evidence to the result.

Question 12 It can provide evidence to solve the question that whether Brigatinib is available to *ALK*-Positive NSCLC.

Article 9

Brigatinib in ALK+ crizotinib-refractory non–small cell lung cancer: Final results of the Phase 1/2 and Phase 2 (ALTA) trials

Question 1 Yes.

To report long-term efficacy and safety results from the final analyses of the phase 1/2 and phase 2 (ALTA) trials of brigatinib, completed >5 years and >4 years, respectively, after the last patients enrolled.

Question 2 Yes.

Question 3 Yes.

Question 4 Yes.

Question 5(a) Can't tell.
Question 5(b) Can't tell.

Question 6(a) Yes.
Question 6(b) Yes.

Question 7 The numerical value of PFS, iPFS, DCR.ORR and iORR.

Question 8 phase 1/2 trial: PFS:16.3(9.2,27.5) ORR:0.79(0.59,0.92) DCR:0.89(0.72,0.98)
ALTA trial: PFS:16.7(11.6,21.4) ORR:0.56(0.47,0.66) DCR: 0.84(0.75,0.90)
iPFS:18.4(12.6,23.9) iORR:0.67(0.41,0.87)

Question 9 Yes.

Question 10 Yes.

Question 11 Can't tell.

There is no available evidence to the result.

Question 12 It can provide evidence to solve the question that whether Brigatinib is available to *ALK*-Positive NSCLC.

Article 10
Brigatinib in Japanese patients with anaplastic lymphoma kinase–positive non–small cell lung cancer:First results from the J-ALTA tyrosine kinase inhibitor-naive expansion cohort

Question 1   Yes.
This primary analysis from the phase 2 J-ALTA study was conducted to evaluate the efficacy and safety of brigatinib in Japanese patients with advanced ALK+ NSCLC who had
not previously been treated with an ALK TKI.

Question 2   Yes.

Question 3   Yes.

Question 4   Yes.

Question 5(a)   Can't tell.
Question 5(b)   Can't tell.

Question 6(a)   Yes.
Question 6(b)   No.
The median PFS can't be reached.

Question 7   The numerical value of iORR ORR and DCR.
Question 8   ORR 0.97(0.84,1)   iORR 0.40(0.05,0.85)   DCR 0.97(0.84,1)

Question 9   Yes.
Question 10   Yes.

Question 11   Can't tell.
There is no available evidence to the result.

Question 12   It can provide evidence to solve the question that whether Brigatinib is available to *ALK*-Positive NSCLC.
